# Effects of apparent temperature on daily outpatient and inpatient visits for cause-specific respiratory diseases in Ganzhou, China: a time series study

**DOI:** 10.1265/ehpm.23-00188

**Published:** 2024-03-22

**Authors:** Mengxia Qing, Yanjun Guo, Yuxin Yao, Chuanfei Zhou, Dongming Wang, Weihong Qiu, You Guo, Xiaokang Zhang

**Affiliations:** 1 Department of Occupational & Environmental Health, School of Public Health, Tongji Medical College, Huazhong University of Science and Technology, Wuhan, Hubei 430030, China; 2 Key Laboratory of Environment and Health, Ministry of Education & Ministry of Environmental Protection, and State Key Laboratory of Environmental Health (Incubating), School of Public Health, Tongji Medical College, Huazhong University of Science and Technology, Wuhan, Hubei 430030, China; 3 First Affiliated Hospital, Gannan Medical University, Ganzhou, China; 4 Key Laboratory of Prevention and Treatment of Cardiovascular and Cerebrovascular Diseases, Ministry of Education, Gannan Medical University, Ganzhou, China; 5 School of Public Health and Health Management, Gannan Medical University, Ganzhou, China

**Keywords:** Apparent temperature, Respiratory diseases, Respiratory tract infection, Outpatient and inpatient visits, Time-series analysis, Distributed lag nonlinear models

## Abstract

**Background:**

Non-optimum temperatures are associated with increased risk of respiratory diseases, but the effects of apparent temperature (AT) on respiratory diseases remain to be investigated.

**Methods:**

Using daily data from 2016 to 2020 in Ganzhou, a large city in southern China, we analyzed the impact of AT on outpatient and inpatient visits for respiratory diseases. We considered total respiratory diseases and five subtypes (influenza and pneumonia, upper respiratory tract infection (URTI), lower respiratory tract infection (LRTI), asthma and chronic obstructive pulmonary disease [COPD]). Our analysis employed a distributed lag nonlinear model (DLNM) combined with a generalized additive model (GAM).

**Results:**

We recorded 94,952 outpatients and 72,410 inpatients for respiratory diseases. We found AT significantly non-linearly associated with daily outpatient and inpatient visits for total respiratory diseases, influenza and pneumonia, and URTI, primarily during comfortable AT levels, while it was exclusively related with daily inpatient visits for LRTI and COPD. Moderate heat (32.1 °C, the 75.0th centile) was observed with a significant effect on both daily outpatient and inpatient visits for total respiratory diseases at a relative risk of 1.561 (1.161, 2.098) and 1.276 (1.027, 1.585), respectively (both *P* < 0.05), while the results of inpatients became insignificant with the adjustment for CO and O_3_. The attributable fractions in outpatients and inpatients were as follows: total respiratory diseases (24.43% and 18.69%), influenza and pneumonia (31.54% and 17.33%), URTI (23.03% and 32.91%), LRTI (37.49% and 30.00%), asthma (9.83% and 3.39%), and COPD (30.67% and 10.65%). Stratified analyses showed that children ≤5 years old were more susceptible to moderate heat than older participants.

**Conclusions:**

In conclusion, our results indicated moderate heat increase the risk of daily outpatient and inpatient visits for respiratory diseases, especially among children under the age of 5.

**Supplementary information:**

The online version contains supplementary material available at https://doi.org/10.1265/ehpm.23-00188.

## Graphical Abstract

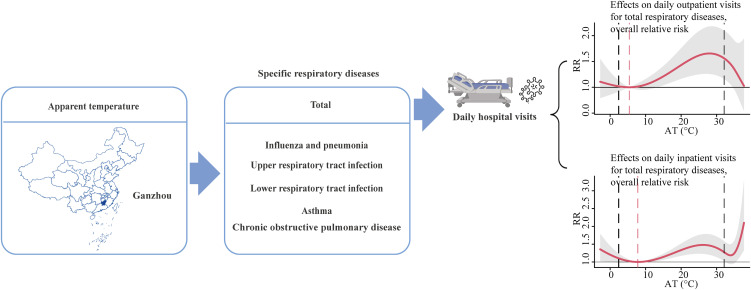

## 1. Introduction

With increasing concerns about anthropogenic greenhouse gas emissions catalyzing the changes in global climate, health consequences arising from temperature have gained significant attention. Previous studies have found temperature adversely affected cardiovascular diseases [1, 2], suicidal behavior [3], overall mortality [4, 5] and other related outcomes [6]. Given that the respiratory system is directly and significantly influenced by temperature, its effects have been of greater concern than other systems. Non-optimum temperatures have been reported to be related to elevating risks of respiratory diseases. There is evidence suggesting that the activity of many respiratory pathogenic microorganisms, which induce most respiratory infections [7], is deeply affected by temperatures [8, 9]. For instance, a time-series analysis in northwest China suggested a reverse J-shaped association between non-optimum temperatures and clinical visits for respiratory diseases [10]. A study conducted in Taiwan reported the adverse effects of non-optimum temperatures on emergency room visits for respiratory diseases [11]. A previous study in 12 European cities has indicated the associations between high temperatures and hospital admissions for respiratory diseases [12]. Turner et al. observed an increased risk of respiratory morbidity related to an increase in ambient temperature on hot days through a systematic review of multiple results [13]. Besides, there are several pieces of research to explore the relationships between asthma and non-optimum temperatures [14, 15]. However, the effects of non-optimum temperatures on other specific respiratory diseases such as influenza and pneumonia, lower respiratory tract infection (LRTI) and others have not been consistently or commonly discussed before.

Further epidemiological research has been conducted to investigate the burden of respiratory mortality attributable to temperature. For example, Fu et al. have found that moderate cold was related to 6.50% mortality from respiratory diseases in Indian [16]. About 8.63% of respiratory mortality was attributed to temperature in a study of five East-Asian countries/regions [17]. The attributable fraction was 10.57% for overall respiratory diseases in main cities in China [18]. Denpetkul et al. found that 3.00% of respiratory mortality in Thailand was due to non-optimum temperatures [19]. And yet, studies focusing on the burden of outpatient and inpatient visits for temperature-related respiratory illnesses are limited.

Although both ambient temperature [20] and apparent temperature (AT) [4] have been widely used to explain the relationships between temperature and health, AT appears to be the most reasonable variable to measure the overall impact of temperature, according to recent research [21]. The comprehensive biometeorological index, AT, is reported to be an effective predictor of heat-related mortality [22], with adjusting for wind speed and relative humidity together.

Therefore, we conducted a time-series analysis in Ganzhou to investigate the associations of AT with daily inpatient and outpatient visits for respiratory diseases and five cause-specific subtypes, including influenza and pneumonia, upper respiratory tract infection (URTI), LRTI, asthma and chronic obstructive pulmonary disease (COPD). Besides, we utilized attributable fractions to quantify the relative contribution of non-optimum ATs to excess daily outpatient and inpatient visits [18].

## 2. Method

### 2.1. Area

This study was performed in Ganzhou (24°29′–27°09′ N; 113°54′–116°38′ E), a southern city in China. Ganzhou is the biggest city in Jiangxi province, with residents reaching 8.97 million on November 1, 2020, among which 2.07 million (23.09%) are ≤14 years old, according to the seventh National Census. It has a subtropical humid monsoon climate, characterized by distinctive seasons, monsoons prevailing in winter and summer, and rainy spring and summer. In 2020, the daily average temperature of Ganzhou ranged from 2.4 °C to 33.5 °C.

### 2.2. Data collection

#### 2.2.1. Inpatient and outpatient visits

Our study was based on daily inpatient and outpatient visits due to respiratory diseases collected from the medical database of the biggest hospital in the city from January 1, 2016 to December 31, 2020. The gender and age of each case were also collected. According to ICD-10 (international classification of diseases, 10th revision) codes, respiratory diseases (J00–J98) were classified into five categories: URTI (J00–J06), influenza and pneumonia (J09–J18), LRTI (J20–J22), COPD (J41–J44) and asthma (J45–J46). The COVID-19 code, U07.1, was not included in the codes for respiratory diseases in our study [23].

#### 2.2.2. Meteorological and air pollutant data

We collected daily meteorological data, including daily mean temperature (T_mean_), daily maximum temperature (T_max_) and daily minimum temperature (T_min_), relative humidity (RH), atmospheric pressure as well as wind speed (WS) from China Meteorological Data Sharing Service Center (http://data.cma.cn/). Daily fine particulate matter (PM_2.5_), inhalable particles (PM_10_), ozone (O_3_), nitrogen dioxide (NO_2_), sulfur dioxide (SO_2_), and carbon monoxide (CO) concentrations were extracted from the National Urban Air Quality Real-Time Release Platform developed by China National Environmental Monitoring Centre (http://www.cnemc.cn/). Missing data of meteorological factors and air pollutants were imputed by an average of the preceding and subsequent seven days.

### 2.3. Temperature exposure assessment

AT was different from ambient temperature and was the comprehensive exposure metric of temperature. It was evaluated based on T_mean_ (°C), RH (%), and WS (m/s) by the following formulas [24]:

$$AT=T_{\text{mean}}+0.33*e-0.70*WS-4.00$$ (a)

$$e=\frac{RH}{100}*6.105*\exp\left(17.27*\frac{T_{\text{mean}}}{237.7+T_{\text{mean}}}\right)$$ (b)

Such that *T_mean_* is mean ambient temperature; *e* represents water vapor pressure (hpa), which can be evaluated by Eq. (b).

### 2.4. Statistical analysis

Using Spearman rank correlation, we evaluated the correlation between meteorological variables and air pollutants.

The distributed lag nonlinear model (DLNM) combined with a Poisson regression was used to estimate the non-linear exposure-response associations of AT and its lagged effects with daily outpatient and inpatient visits since AT was reported to be associated with human health in a non-linear pattern [4]. As the discreteness of the daily outpatient and inpatient visits for respiratory diseases showed over-dispersion, we used the quasi-Poisson method. Following previous investigations [5, 25, 26] and excluding atmospheric pressure, which was strongly related to AT (the absolute value of correlation coefficient was more than 0.60), we adjusted the following covariates:

1. A natural spline function of calendar time with 8 degrees of freedom (df) per year to control the effects of long-term trends of daily hospital visits and the effects of seasonality.
2. Natural spline functions of air pollutants such as PM_2.5_ with 3 df.
3. Indicator variables of the day of the week and holiday.

The cross-basis function conducted by basis functions of non-linear and lagged effects of AT was used to estimate the combined effects of AT in DLNM. The basis functions of exposures were the nature spline functions with three internal knots placed at the 10th, 75th, and 90th AT percentiles. The basis functions of lagged dimension were built with three internal knots placed at equally spaced temperature percentiles (25th, 50th, and 75th). Following previous studies [18, 27], we assumed the hysteresis effects of AT on respiratory diseases were presented for up to 21 days in the present study. The model frame was as follows:

$$\begin{array}{rl} g(\mu_{t}) & =\alpha +cb({AT}_{t,l})+ns({\text{Time}}_{t},8)+ns({PM}_{2.5_{t}},3)\\ & \;+\lambda {\text{dow}}_{t}+\gamma {\text{Holiday}}_{t} \end{array}$$ (c)

Such that *g*() is the link function; *μ_t_* is the expected number of inpatient or outpatient visits for respiratory diseases on day *t*; *α* is the intercept; *cb*(*AT_t_*_,_*_l_*) is the crossbasis function of AT obtained through the DLNM; *l* is the maximum lag days; *ns*() is the natural spline function; *dow_t_* means the term of the day of week; *Holiday_t_* means the term of public holidays.

We used AT corresponding to the minimum or relative minimum daily patient visits for respiratory diseases as the optimum AT, or AT close to optimum AT (to observe better the effects of moderate cold and moderate heat AT on diseases) as the reference AT (the defined optimum AT) to calculate the relative risk (RR) of relative AT. We categorized the AT range into extreme cold, moderate cold, comfortable, moderate heat, and extreme heat. The centiles of five classifications of AT ranges were <2.5th centile, 2.5th centile up to the reference AT, the reference AT up to the 75.0th centile, the 75.0th centile up to the 97.5th centile, and >97.5th centile, respectively [5, 18, 28]. We assessed the overall and delayed effects of AT on the risk of daily outpatient and inpatient visits for respiratory diseases, specifically concentrating on two specific temperature percentiles: the 2.5th percentile at 2.4 °C and the 75.0th percentile at 32.1 °C. They are both lowest temperature of the moderate range, defined as moderate cold and moderate heat in the following analyses (excluding attributable risk fractions). We selected these percentiles because of those representative temperature points demonstrated a statistically significant associations with respiratory diseases risks within the moderate range. Moderate temperatures, which are more common than extreme temperatures [18, 29], particularly in Ganzhou, were the primary focus of our study. A recent study has found that among moderate and extreme temperatures, moderate temperatures had the greatest impact on mortality [4]. The Quasi-Akaike information criterion was conducted to estimate the model’s best df for time trend terms. Of df for air pollutants in our analysis, the selection of three was based on previous studies [30].

To evaluate the relative contribution of AT to respiratory disease burdens, we calculated the fractions of attributable daily outpatient and inpatient visits during the present and lagged days using the method from forward perspectives in the DLNM framework according to the study of Gasparrini et al. [31]. The overall attributable fraction was a sum of the contribution from all the days of exposure using optimum AT (or reference AT described above) as the reference. To evaluate the attributable fractions associated with extreme cold, moderate cold, comfortable, moderate heat and extreme heat AT, we summed the data of days with relevant AT ranges according to specific centiles of AT distribution. Empirical confidence intervals (eCI) were derived by calculating overall and for separated components by simulating samples from specific distributions [31].

Stratified analyses by gender (male/female) and age (≤5, >5 to ≤14, >14 to ≤44, >44 to ≤65/ >65) were conducted to test the reliability of results between different subgroups. As respiratory tract infection was the leading cause of death of children under five years old [32], we set one of the cut-off points of age at five years old. The difference between the two estimates of subgroups was indicated by the *P* value calculated through Z-test [33].

### 2.5. Sensitivity analyses

Sensitivity analyses included additionally adjusting for other air pollutants, including CO and O_3_ with 3 df, sunshine time with 3 df, and using ambient temperature rather than AT as exposure to analyze relationships between temperature and daily outpatient and inpatient visits for respiratory diseases. To control for the potential impacts of RH and WS, we adjusted those two confounders while fitting the model for ambient temperature. To avoid collinearity, air pollutants with a strong correlation (r > 0.6) were not included as adjustment variables [34]. In the study, we considered two-tailed *P* < 0.05 statistically significant. All statistical analyses were conducted by R software (version 4.1.2) using “dlnm” package.

## 3. Results

### 3.1. Data description

Table 1 presents the characteristics of daily inpatient and outpatient visits for respiratory diseases and five cause-specific subtypes and meteorological variables in Ganzhou, 2016–2020. The average daily AT was 22.0 °C, ranging from −2.9 °C to 37.8 °C. The total numbers of registered outpatient and inpatient visits for respiratory diseases were 94,952 and 72,410 with daily averages of 51.9 and 39.6, respectively. For outpatients, there were 41,953 (44.2%) female patients and 22,184 (23.4%) patients aged ≤5 years. For inpatients, there were 22,824 (31.5%) female patients and 20,071 (27.7%) patients aged ≤5 years.

**Table 1:** Distribution of daily hospital visits for respiratory diseases and meteorological variables in Ganzhou, 2016–2020

**Variables**	**Number (%)**	**Mean ± SD**	**Minimum** **value**	**P25**	**P50**	**P75**	**Maximum** **value**
Meteorological variables							
AT (°C)		22.0 ± 10.6	−2.9	12.8	23.0	32.1	37.8
T_mean_ (°C)		20.6 ± 8.1	1.0	13.7	21.7	27.8	33.5
Relative humidity (%)		75.2 ± 12.0	35.5	66.3	75.5	85.0	99.0
No. of daily outpatient visits for respiratory diseases							
Total	94,952 (100.0)	51.9 ± 31.3	1	33	46	60	210
Male	52,999 (55.8)	29.0 ± 18.1	0	18	25	35	117
Female	41,953 (44.2)	22.9 ± 14.2	0	14	20	27	93
Age, years							
≤5	22,184 (23.4)	12.1 ± 16.2	0	4	7	13	116
>5 to ≤14	7,397 (7.8)	4.1 ± 4.8	0	1	3	5	57
>14 to ≤44	24,891 (26.2)	13.6 ± 8.6	0	8	12	17	73
>44 to ≤65	22,879 (24.1)	12.5 ± 7.0	0	7	12	17	48
>65	17,601 (18.5)	9.6 ± 6.2	0	5	9	14	39
Influenza and pneumonia	8,961 (9.4)	4.9 ± 3.3	0	2	4	7	21
URTI	40,050 (42.2)	21.9 ± 25.4	1	9	13	21	144
LRTI	5,509 (5.8)	3.0 ± 4.8	0	0	1	4	47
Asthma	28,827 (30.4)	15.8 ± 11.0	0	7	14	24	52
COPD	11,605 (12.2)	6.4 ± 5.2	0	2	5	9	35
No. of daily inpatient visits for respiratory diseases							
Total	72,410 (100.0)	39.6 ± 15.9	5	28	37	49	108
Male	49,586 (68.5)	27.1 ± 11.3	3	19	26	34	79
Female	22,824 (31.5)	12.5 ± 6.0	0	8	12	16	38
Age							
≤5	20,071 (27.7)	11.0 ± 6.0	0	7	10	14	46
>5 to ≤14	4,659 (6.4)	2.6 ± 2.1	0	1	2	4	15
>14 to ≤44	4,868 (6.7)	2.7 ± 2.0	0	1	2	4	11
>44 to ≤65	19,103 (26.4)	10.5 ± 6.4	0	6	9	14	38
>65	23,709 (32.7)	13.0 ± 6.8	0	8	12	17	51
Influenza and pneumonia	21,452 (29.6)	7.7 ± 5.3	0	4	6	10	37
URTI	16,073 (22.2)	8.8 ± 4.1	0	6	8	11	31
LRTI	4,724 (6.5)	2.6 ± 2.1	0	1	2	4	14
Asthma	5,588 (7.7)	3.1 ± 2.0	0	2	3	4	13
COPD	24,573 (33.9)	13.5 ± 6.5	0	9	13	18	42

**Abbreviations:** AT, apparent temperature; SD, standard deviation; T_mean_, daily average temperatures; COPD, chronic obstructive pulmonary disease; URTI, upper respiratory tract infection; LRTI, lower respiratory tract infection.

Summary statistics on meteorological variables and air pollutants from 1 January 2016 to 31 December 2020 in Ganzhou are shown in Table S1. Time-series distributions, which revealed temporal trends of daily outpatient and inpatient visits for respiratory diseases, temperature indexes, meteorological variables, and air pollutants in Ganzhou from 2016 to 2020 are displayed in Fig. S1 and Fig. S2. The correlation coefficients between meteorological variables and air pollutants using Spearman rank correlation are shown in Fig. S3.

### 3.2. Associations between AT and daily outpatient and inpatient visits for respiratory diseases

**Fig. 1:**
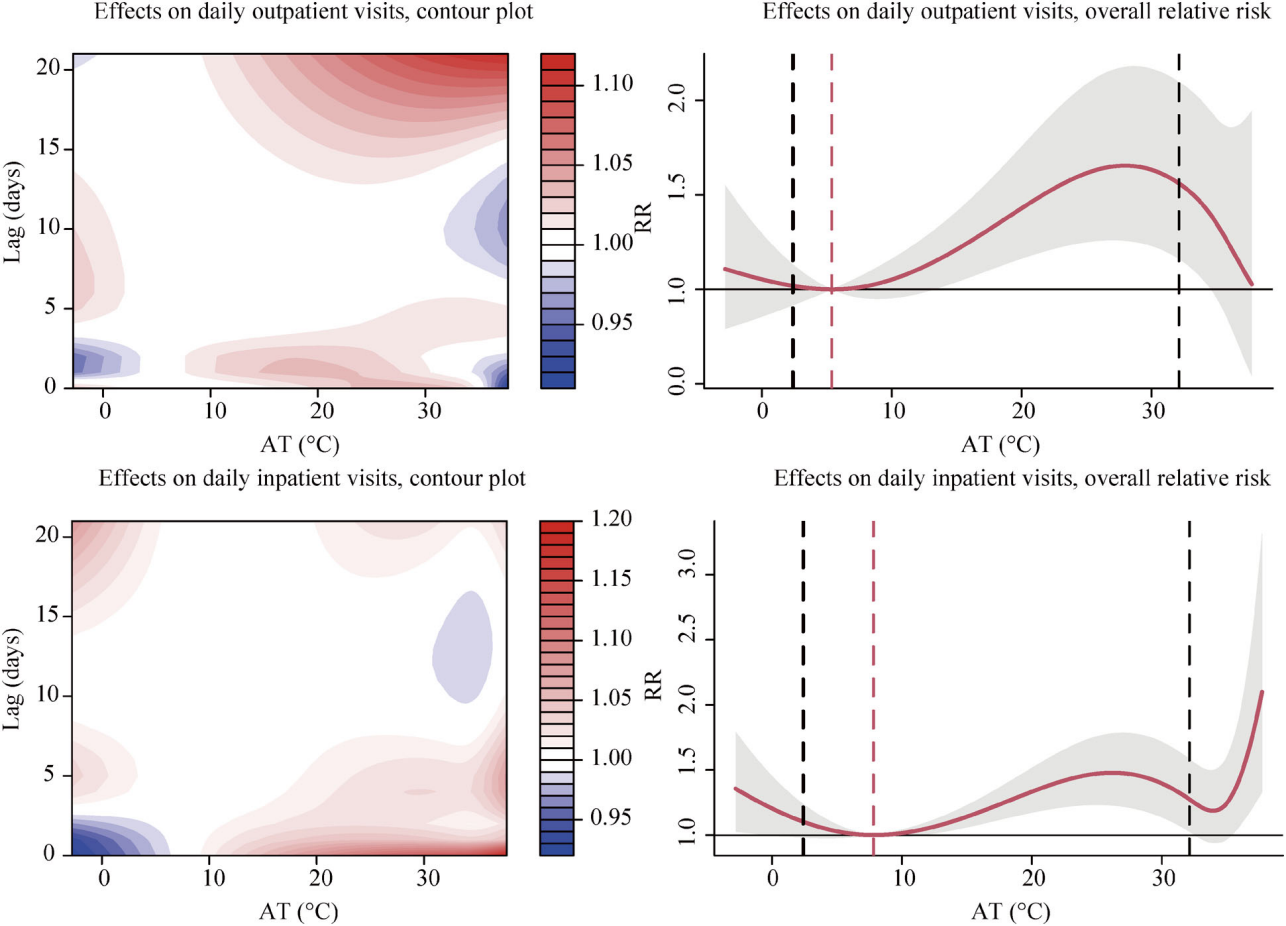
Exposure-response relationships between AT and risk of daily hospital visits for total respiratory diseases AT, apparent temperature; RR, relative risk. Solid lines = mean RR (AT v the reference AT); shaded areas = 95% confidence intervals; red dashed lines = reference AT (5.4 °C in outpatients and 7.8 °C in inpatients); black dashed lines = moderate cold (2.5th, 2.4 °C) and moderate heat (75.0th, 32.1 °C), respectively.

The exposure-response relationships between AT and the risk of daily outpatient and inpatient visits for total respiratory diseases and the delayed effects of AT are presented in Fig. 1. There was a significantly positive association between AT and the risk of daily visits for total respiratory diseases in both outpatients and inpatients when AT was higher than the optimum level (5.4 °C in outpatients and 7.8 °C in inpatients) in most of the comfortable ATs. The overall exposure-response relationships between AT and outpatient and inpatient visits due to five cause-specific respiratory diseases are shown in Fig. S4. There was a non-linear relationship between AT and daily outpatient visits for influenza and pneumonia and URTI in most of the comfortable ATs. Of daily hospitalizations for influenza and pneumonia, URTI, LRTI, and COPD, the curve showed a non-linear rise in most of the comfortable ATs.

Moderate heat (75.0th, 32.1 °C) presented significant overall effects on both daily outpatient and inpatient visits for total respiratory diseases and URTI (Table 2). In contrast, the overall effects of moderate cold (2.5th, 2.4 °C) on total respiratory diseases and cause-specific subtypes were insignificant.

**Table 2:** Relative risks of daily hospital visits for specific respiratory diseases associated with non-optimum ATs

**Variables**	**Reference** **AT**	**Relative risk (95% CI)**	

		**Moderate cold** **(2.5th, 2.4 °C)**	**Moderate heat** **(75.0th, 32.1 °C)**
Daily outpatient visits for respiratory diseases			
Total	5.4	1.018 (0.916, 1.132)	**1.561 (1.161, 2.098)**
Influenza and pneumonia	4.0	0.967 (0.877, 1.065)	1.647 (0.905, 3.000)
URTI	9.0	1.262 (0.958, 1.663)	**1.615 (1.129, 2.309)**
LRTI	9.4	1.566 (0.875, 2.802)	1.765 (0.666, 4.681)
Asthma	4.0	0.981 (0.911, 1.056)	1.082 (0.697, 1.680)
COPD	4.0	0.906 (0.799, 1.027)	1.600 (0.856, 2.990)
Daily inpatient visits for respiratory diseases			
Total	7.8	1.103 (0.981, 1.239)	**1.276 (1.027, 1.585)**
Influenza and pneumonia	7.4	1.087 (0.921, 1.284)	1.209 (0.890, 1.642)
URTI	8.7	1.195 (0.963, 1.482)	**2.007 (1.353, 2.976)**
LRTI	6.7	1.078 (0.794, 1.464)	1.790 (0.890, 3.602)
Asthma	10.0	1.137 (0.789, 1.637)	0.964 (0.537, 1.732)
COPD	9.0	1.064 (0.874, 1.295)	1.073 (0.777, 1.481)

**Abbreviations:** AT, apparent temperature; CI, confidence interval; COPD, chronic obstructive pulmonary disease; URTI, upper respiratory tract infection; LRTI, lower respiratory tract infection.

The statistically significant relative risks are highlighted in bold.

**Fig. 2:**
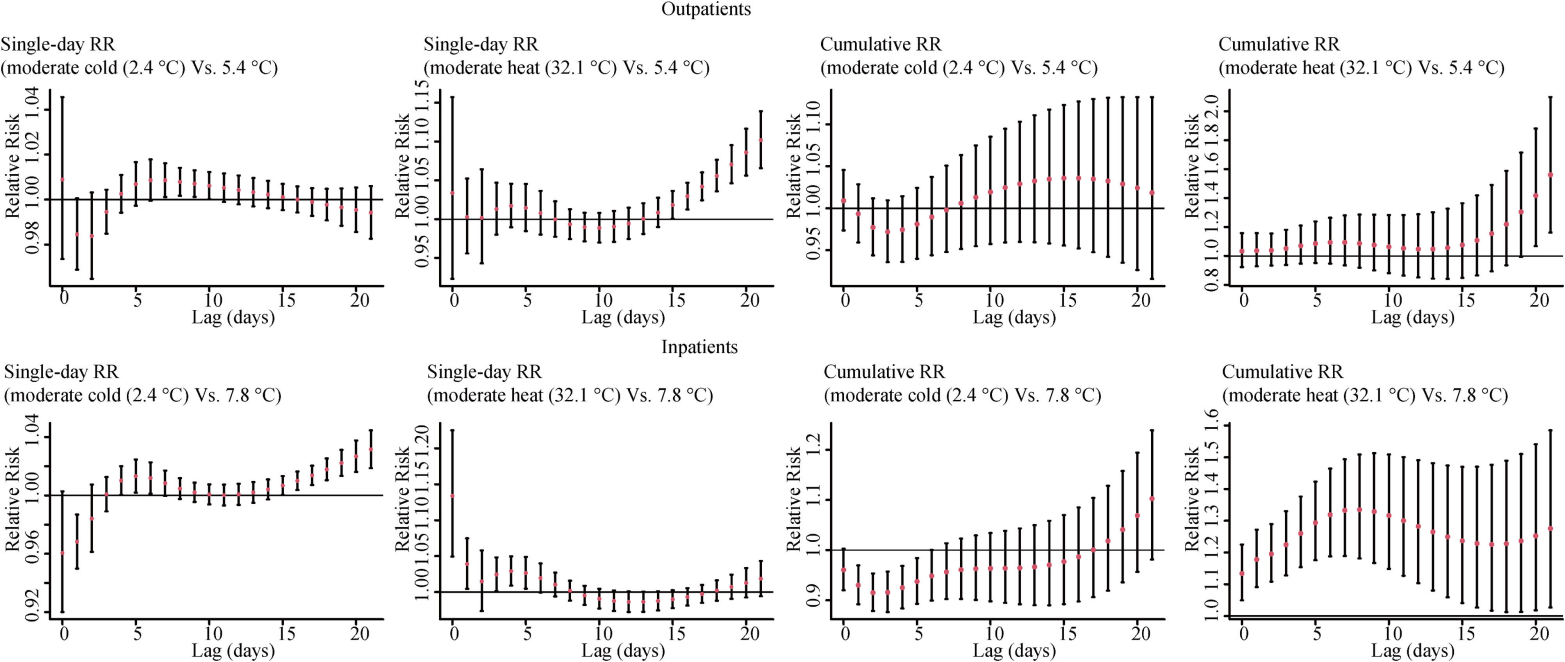
Lag-effects of moderate cold and moderate heat on daily hospital visits for total respiratory diseases* *Moderate cold (2.4 °C) and moderate heat (32.1 °C) were the 2.5th and 75.0th percentiles of AT, respectively. The reference was the optimum AT of each group.

Effects of AT on daily outpatient and inpatient visits for respiratory diseases showed different lag patterns. Figure 2 illustrates the single lag and cumulative lag effects of moderate cold (2.5th, 2.4 °C) and moderate heat (75.0th, 32.1 °C) on daily inpatient and outpatient visits for total respiratory diseases. For moderate cold, with reference to the defined optimum AT (5.4 °C in outpatients and 7.8 °C in inpatients), single lag risky effects on total respiratory diseases appeared at lag7 to lag10 in outpatients and appeared at lag4 to lag7 in inpatients. For moderate heat, risky effects appeared at ∼lag15 and gradually increased as the lag days extended in outpatients and appeared at lag0 and lasted for five days except for lag3 in inpatients. It showed the cumulative lag effects at lag020 and lag021 in outpatients and harmful cumulative lag effects at lag01 to lag021 in inpatients. The results of lag effects of AT on daily hospital visits for five subtypes are presented in Fig. S5 and Fig. S6.

### 3.3. Stratification analysis

The trends of the overall exposure-response relationship between AT and total respiratory diseases were similar between genders in both outpatient and inpatient analyses (Fig. S7). In contrast, the onset of diseases for females and males was affected by moderate heat in outpatient analyses and females were more vulnerable to moderate heat than males, although the difference was not statistically significant (*P* < 0.05, *P* for Z-test > 0.05) (Table S2). There was no significant effect modification of gender for inpatient groups (*P* for Z-test > 0.05) (Fig. S7, Table S2). In terms of age, participants ≤5 years old were more susceptible to moderate heat when compared with other age subgroups in both outpatient and outpatient analyses (*P* for Z-test > 0.05) (Fig. S7, Table S2). The results of lag effects of moderate AT on daily hospital visits for total respiratory diseases, stratified by gender and age, are presented in Fig. S8 and Fig. S9.

### 3.4. Risks of daily outpatient and inpatient visits for respiratory diseases attributable to ATs

To evaluate the excess risks of daily outpatient and inpatient visits for total respiratory diseases and five cause-specific subtypes attributable to non-optimum ATs, we calculated attributable fractions, which were associated with overall days and five subsets of days of relevant AT ranges (Table 3). The comfortable ATs (ranging from the reference AT to 32.1 °C) were responsible for most daily outpatient and inpatient visits for total respiratory diseases attributable to non-optimum temperatures compared with other temperature ranges. Besides, the moderate heat ATs (32.1 to 36.3 °C) made a robust contribution to excessive daily outpatient and inpatient visits for total respiratory diseases, although the estimate was not statistically significant in inpatients.

**Table 3:** Attributable fractions of daily hospital visits for cause-specific respiratory diseases associated with non-optimum ATs

**Variables**	**Reference** **AT**	**Attributable fraction (%; 95% eCI)**					

		**Overall**	**Extreme cold**	**Moderate cold**	**Comfortable**	**Moderate heat**	**Extreme heat**
Daily outpatient visits for respiratory diseases							
Total	5.4	**24.43 (8.61, 36.52)**	0.10 (−0.36, 0.47)	0.02 (−0.13, 0.15)	**17.82 (8.88, 25.12)**	**6.28 (0.08, 10.48)**	0.21 (−1.06, 0.91)
Influenza and pneumonia	4.0	**31.54 (7.09, 49.63)**	−0.24 (−1.30, 0.50)	−0.03 (−0.12, 0.06)	**24.49 (6.93, 36.16)**	6.49 (−5.55, 12.65)	0.84 (−1.65, 1.83)
URTI	9.0	**23.03 (2.84, 37.53)**	0.46 (−0.05, 0.76)	0.48 (−0.48, 1.28)	**21.77 (13.43, 28.73)**	2.44 (−7.28, 9.46)	−2.11 (−5.79, −0.15)
LRTI	9.4	37.49 (−5.30, 54.72)	1.30 (−0.37, 2.05)	1.26 (−1.39, 3.34)	**25.41 (1.75, 38.87)**	8.59 (−10.17, 14.49)	0.93 (−2.39, 1.40)
Asthma	4.0	9.83 (−20.44, 30.06)	−0.13 (−0.72, 0.36)	−0.02 (−0.08, 0.05)	6.75 (−12.17, 20.00)	2.48 (−8.54, 9.44)	0.76 (−0.78, 1.47)
COPD	4.0	30.67 (−6.01, 50.13)	−0.68 (−1.91, 0.11)	−0.08 (−0.2, 0.02)	20.31 (−4.14, 34.78)	9.96 (−2.89, 16.46)	1.17 (−0.48, 1.71)
Daily inpatient visits for respiratory diseases							
Total	7.8	**18.69 (8.60, 27.24)**	**0.40 (0.03, 0.74)**	0.21 (−0.16, 0.57)	**12.91 (6.50, 18.08)**	4.17 (−0.38, 7.88)	**1.02 (0.39, 1.44)**
Influenza and pneumonia	7.4	**17.33 (2.21, 29.43)**	0.35 (−0.26, 0.82)	0.15 (−0.3, 0.58)	**13.16 (4.16, 19.97)**	2.81 (−5.23, 8.16)	0.86 (−0.24, 1.46)
URTI	8.7	**32.91 (20.17, 42.82)**	0.66 (−0.06, 1.20)	0.49 (−0.31, 1.28)	**18.35 (9.69, 25.17)**	**11.57 (6.23, 15.01)**	**1.82 (1.11, 2.18)**
LRTI	6.7	**30.00 (0.45, 46.53)**	0.38 (−1.22, 1.23)	0.14 (−0.74, 0.87)	**22.16 (4.71, 33.82)**	6.66 (−5.43, 12.33)	0.66 (−1.74, 1.31)
Asthma	10.0	3.39 (−38.56, 27.77)	0.47 (−1.05, 1.36)	0.42 (−1.06, 1.57)	2.36 (−21.62, 16.30)	−0.51 (−19.08, 8.96)	0.65 (−2.18, 1.68)
COPD	9.0	10.65 (−8.06, 24.29)	0.27 (−0.44, 0.77)	0.08 (−0.61, 0.69)	9.70 (−0.11, 16.95)	0.07 (−8.63, 6.51)	0.52 (−0.90, 1.37)

**Abbreviations:** AT, apparent temperature; eCI, 95% empirical confidence intervals; COPD, chronic obstructive pulmonary disease; URTI, upper respiratory tract infection; LRTI, lower respiratory tract infection.

Extreme cold ATs range from −2.9 to 2.4 °C, moderate cold ATs range from 2.4 °C to the reference AT, comfortable ATs range from the reference AT to 32.1 °C, extreme heat ATs range from 36.3 to 37.8 °C, and moderate heat ATs range from 32.1 to 36.3 °C. The statistically significant relative risks are highlighted in bold.

### 3.5. Sensitivity analysis

Results of sensitivity analyses in the associations of AT with total respiratory diseases and five cause-specific subtypes are shown in Fig. S10 to Fig. S14 and Table S3 to Table S7. Additional adjustment for sunshine time, CO or O_3_ did not significantly modify the associations between AT and outpatient and inpatient visits for total respiratory diseases and cause-specific subtypes. The risky effects of moderate heat on daily inpatient visits for total respiratory diseases were insignificant with the adjustment for CO and O_3_, although the trends remained similar. Using ambient temperature rather than AT as exposure, the significant effects of moderate cold (5.4 °C) on both inpatient and outpatient visits for URTI, and on inpatient visits for total respiratory diseases were observed.

## 4. Discussion

Our findings demonstrated a non-linear association between AT and daily outpatient and inpatient visits for respiratory diseases in most of the comfortable temperatures. Moderate heat was associated with increased risks of daily outpatient and inpatient visits for total respiratory diseases and URTI. In addition, more outpatient and inpatient visits for respiratory disease were attributable to comfortable and moderate heat ATs rather than moderate cold ATs, when compared to the optimum AT. Significant age modifications were shown in our results.

A number of epidemiological studies have investigated the harmful effects of non-optimum temperatures on respiratory diseases [10, 18, 30, 35, 36]. Those researches all emphasized the risky effects of extreme temperatures, which was not the main concern of our study. The weather characteristic in Ganzhou is different from other climatic zones, emphasizing the harmful effects of moderate temperatures [4]. In our study, the defined comfortable and moderate heat ATs were mainly responsible for daily hospital visits burden for respiratory diseases. Moderate temperatures, including the seasonal transitions that coincide with a high prevalence of respiratory diseases, may be responsible for the observed adverse effects [4]. Increased risks of hospital visits for respiratory diseases corresponding to stronger effects of moderate temperatures emphasize the importance of improving awareness of traditionally considered comfortable and moderate temperatures, not only extreme temperatures. Taking the coastal climate of the city into consideration, we summarized the non-accidental mortality risk attributable to AT in Busan and Incheon with that in Seoul, Daegu, Gwangju, and Daejeon in Korea [4]. In contrast to our study, most temperature-attributable deaths were caused by cold, and only a small proportion by heat in those coastal and non-coastal cities.

Several explanations may contribute to the effects of moderate cold on respiratory diseases shown in this analysis. Firstly, the external cold temperature stimulates the stress response of respiratory system, which makes it more sensitive to pathogenic microorganisms, increasing risks of respiratory tract infections. Secondly, cold damage the movement of bronchial cilia and weaken the phagocytosis of alveolar macrophages, reducing immunity of the human [8, 37]. Thirdly, cumulative exposure to cold weather elevates inflammation marker levels [38], for example, the numbers of granulocytes and macrophages in lower airways [39], indicating that cold may lead to inflammation response. Finally, previous studies suggested that cold stress induced the release of stress hormones such as epinephrine, norepinephrine and cortisol [40–42]. These physiological changes may trigger respiratory infections. In addition, pathogenic microorganisms are easier to live in the outside environment in cold season, resulting in more infection opportunities. In agreement with findings from previous studies [30, 43], the lag pattern of moderate heat in inpatients of our research was characterized as acute. The mechanisms of the effects of heat AT on respiratory diseases may include restraining the release of proinflammatory cytokines [44, 45], inducing oxidative stress [46, 47], for example, the increase in catalase and superoxide dismutase [48], elevating the adrenocorticotropic hormone and cortisol [45].

Children (≤5 years old) were more vulnerable to non-optimal temperature for respiratory diseases than other older subgroups in our study. The high prevalence of respiratory tract infections among children younger than five years old (among which the viral positivity rate of acute respiratory infections is 46.9% [49]) may partially explain the difference. The vulnerability of young children to non-optimal temperatures may also be due to their greater metabolic rate [30] and respiratory system of young children is more fragile [9].

We compared the effects of temperatures on respiratory diseases using identical percentiles for AT and ambient temperature. Ambient temperatures demonstrated higher risky effects of moderate cold on both inpatient and outpatient visits for URTI and on inpatient visits for total respiratory diseases than AT in our results. According to Ho et al., AT is a better predictor of heat-related damage and indoor temperature than ambient temperature [50, 51]. The AT commonly exhibits variations of several degrees in comparison to the ambient temperature. A spatial temperature study has proved there were significant spatial differences in distribution between air temperature and AT [50]. Utilizing AT for mapping allows for a more accurate quantification of heat exposure [50]. As the heat temperature induced body damage depending on perception of temperature and thermal stress, there was a necessity to employ variables better describing the perception compared to ambient temperature, such as AT [21]. Because humidity and wind speed both influence heat exchange efficiency, AT, a comprehensive index of ambient temperature, relative humidity and wind speed, reflects human somatosensory more objectively than ambient temperature [4]. The AT has been observed to more objectively reflect human thermal perception compared to the mere ambient temperature in the European study [52]. He et al found AT exposure were more effective than ambient temperature in influencing variability of asthma symptoms [53]. However, the effectiveness of ambient temperature and AT of the perception of cold stress was not well established. A study conducted in Iran found preterm birth risk associated with AT peaks in both extreme hot and cold conditions compared to maximum and mean temperatures [54]. The other study found no significant overall difference in the impact on semen quality between exposure to AT and exposure to ambient temperature [55]. Thus, more relevant studies are warranted.

Our study has some merits. Firstly, we identified non-optimum ATs increased risks of daily inpatient visits for total respiratory diseases, influenza and pneumonia, URTI, LRTI, and COPD in Ganzhou, China. The stratified analyses revealed the effect modifications by age. We also calculated the attributable fractions to evaluate the excess risks of disease burden due to AT. Secondly, our study included data derived from outpatients and inpatients, differed in volume and severity, with a relatively large sample size, enabling us to investigate with differently sensitive outcomes. Finally, sensitivity analyses included additionally adjusting for other air pollutants, including CO and O_3_ with 3 df, sunshine time with 3 df, as well as using ambient temperature rather than AT as exposure were performed to test the robustness of our findings. We also acknowledge our limitations. First, the causality between the temperature index and respiratory diseases could not be testified in our ecological study. Second, we used meteorological factor data obtained from fixed monitoring stations rather than individual-level data. The effects of meteorological factors may be underestimated as measurement errors might exist in our data [56]. However, research using data obtained from fixed monitoring stations has been widely accepted, making those results comparable. Thirdly, we did not explore how COVID-19 influence the associations between AT and respiratory diseases, as incidence of respiratory diseases might falsely fluctuate due to COVID-19. Finally, the study only collected data from one hospital in Ganzhou, which may underestimate the effects of AT. Extrapolation should be conducted and interpreted with caution.

## 5. Conclusion and implications

Our study revealed that moderate heat was associated with an increased risk of daily inpatient visits for various respiratory diseases, including total respiratory diseases and URTI in Ganzhou, China. These findings emphasize the significance of non-optimum ATs and offer insights for enhancing local public health policy practices.

## Abbreviations

AT: apparent temperature
COPD: chronic obstructive pulmonary disease
URTI: upper respiratory tract infection
LRTI: lower respiratory tract infection
CI: confidence interval
df: degrees of freedom
DLNM: distributed lag nonlinear model
GAM: generalized additive model
eCI: empirical confidence intervals
ICD: International Classification of Diseases
PM_2.5_: fine particulate matter
PM_10_: inhalable particles
O_3_: ozone
NO_2_: nitrogen dioxide
SO_2_: sulfur dioxide
CO: carbon monoxide
RH: relative humidity
RR: relative risk
SD: standard deviation
T_max_: daily maximum temperature
T_mean_: daily mean temperature
T_min_: daily minimum temperature
WS: wind speed

## Supplementary information

**Additional file 1:** **Fig. S1** Time-series distributions of daily hospital visits for cause-specific respiratory diseases in Ganzhou, 2016–2020. **Fig. S2** Time-series distributions of meteorological variables and concentrations of air pollutants in Ganzhou, 2016–2020. **Fig. S3** Spearman’s correlation of air pollutants and meteorological variables. **Fig. S4** Exposure-response relationships between AT and risk of daily hospital visits for five cause-specific respiratory diseases*. **Fig. S5** Lag-effects of moderate cold and moderate heat on daily outpatient visits for cause-specific respiratory diseases*. **Fig. S6** Lag-effects of moderate cold and moderate heat on daily inpatient visits for cause-specific respiratory diseases*. **Fig. S7** Exposure-response relationships between AT and risk of daily hospital visits for total respiratory diseases*. **Fig. S8** Lag-effects of moderate cold and moderate heat on daily outpatient visits for total respiratory diseases*. **Fig. S9** Lag-effects of moderate cold and moderate heat on daily inpatient visits for total respiratory diseases*. **Fig. S10** Exposure-response relationships between AT and risk of daily hospital visits for respiratory diseases: sensitivity analyses*. **Fig. S11** Exposure-response relationships between AT and risk of daily hospital visits for respiratory diseases: sensitivity analyses*. **Fig. S12** Exposure-response relationships between AT and risk of daily hospital visits for respiratory diseases: sensitivity analyses*. **Fig. S13** Exposure-response relationships between AT and risk of daily hospital visits for respiratory diseases: sensitivity analyses*. **Fig. S14** Exposure-response relationships between AT and risk of daily hospital visits for respiratory diseases: sensitivity analyses*. **Table S1** Summary statistics on meteorological variables and air pollutants in Ganzhou, 2016–2020. **Table S2** Relative risks of daily hospital visits for total respiratory diseases associated with non-optimum ATs*. **Table S3** Relative risks of daily hospital visits for respiratory diseases associated with non-optimum ATs: sensitivity analyses*. **Table S4** Relative risks of daily hospital visits for respiratory diseases associated with non-optimum ATs: sensitivity analyses*. **Table S5** Relative risks of daily hospital visits for respiratory diseases associated with non-optimum ATs: sensitivity analyses*. **Table S6** Relative risks of daily hospital visits for respiratory diseases associated with non-optimum ATs: sensitivity analyses*. **Table S7** Relative risks of daily hospital visits for respiratory diseases associated with non-optimum ambient temperatures: sensitivity analyses*.

## Declarations

### Availability of data and materials

Meteorological data can be accessed from the China Meteorological Data Sharing Service System (http://data.cma.cn/). Air pollution data used in this study can be obtained from China National Environmental Monitoring Centre (http://www.cnemc.cn/). The summarized health data that support the findings of this study are available from the corresponding author, [YJG, YG and XKZ], upon reasonable request.
