# Supplementary material for: Effects of apparent temperature on daily outpatient and inpatient visits for cause-specific respiratory diseases in Ganzhou, China: a time series study
Source: Environ Health Prev Med. 2024 Mar 22;29:20. doi: 10.1265/ehpm.23-00188 (PMC10965414; doi:10.1265/ehpm.23-00188)

**Supplementary graphs and tables**

**Effects of apparent temperature on daily outpatient and inpatient visits for cause-specific respiratory diseases in Ganzhou, China: A time series study**

**Fig. S1 Time-series distributions of daily hospital visits for cause-specific respiratory diseases in Ganzhou, 2016–2020**

COPD, chronic obstructive pulmonary disease; URTI, upper respiratory tract infection; LRTI, lower respiratory tract infection.

**Fig. S2 Time-series distributions of meteorological variables and concentrations of air pollutants in Ganzhou, 2016–2020**

T_max_, daily maximum temperatures; T_min_, daily minimum temperatures; T_mean_, daily average temperatures; AT, apparent temperature; PM_2.5_, fine particulate matter; PM_10_, inhalable particles; O_3_, ozone; NO_2_, nitrogen dioxide; SO_2_, sulfur dioxide; CO, carbon monoxide; WS, wind speed; RH, relative humidity.

**Fig. S3 Spearman’s correlation of air pollutants and meteorological variables**

PM_2.5_, fine particulate matter; T_max_, daily maximum temperatures; T_min_, daily minimum temperatures; T_mean_, daily average temperatures; AT, apparent temperature; PM_2.5_, fine particulate matter; PM_10_, inhalable particles; O_3_, ozone; NO_2_, nitrogen dioxide; SO_2_, sulfur dioxide; CO, carbon monoxide; **P* <0.05.

**Fig. S4 Exposure-response relationships between AT and risk of daily hospital visits for five cause-specific respiratory diseases***
*We excluded data points where the ATs exceeded the 97.5th percentile while plotting the curve of LRTI in outpatients due to the limited amount of data available for those ATs.

AT, apparent temperature; RR, relative risk; COPD, chronic obstructive pulmonary disease; URTI, upper respiratory tract infection; LRTI, lower respiratory tract infection. Solid lines=mean RR (AT v the reference AT); shaded areas=95% confidence intervals; red dashed lines=reference AT (Outpatients: 4.0 °C for influenza and pneumonia, 9.0 °C for URTI, 9.4 °C for LRTI, 4.0 °C for asthma, 4.0 °C for COPD; Inpatients: 7.4 °C for influenza and pneumonia, 8.7 °C for URTI, 6.7 °C for LRTI, 10.0 °C for asthma, 9.0 °C for COPD); black dashed lines=moderate cold (2.5th, 2.4 °C) and moderate heat (75.0th, 32.1 °C), respectively.

**Fig. S5 Lag-effects of moderate cold and moderate heat on daily outpatient visits** **for cause-specific respiratory diseases***

*Moderate cold (2.4 °C) and moderate heat (32.1 °C) were the 2.5th and 75.0th percentiles of AT, respectively. The reference was the optimum AT of each group.
AT, apparent temperature; RR, relative risk; COPD, chronic obstructive pulmonary disease; URTI, upper respiratory tract infection; LRTI, lower respiratory tract infection.

**Fig. S6 Lag-effects of moderate cold and moderate heat on daily inpatient visits for cause-specific respiratory diseases***

*Moderate cold (2.4 °C) and moderate heat (32.1 °C) were the 2.5th and 75.0th percentiles of AT, respectively. The reference was the optimum AT of each group.
AT, apparent temperature; RR, relative risk; COPD, chronic obstructive pulmonary disease; URTI, upper respiratory tract infection; LRTI, lower respiratory tract infection.

**Fig. S7 Exposure-response relationships between AT and risk of daily hospital visits** **for total respiratory diseases***

*This is a subgroup analysis which is stratified by gender, age.
AT, apparent temperature; RR, relative risk; COPD, chronic obstructive pulmonary disease; URTI, upper respiratory tract infection; LRTI, lower respiratory tract infection. Solid lines=mean RR (AT v the reference AT); shaded areas=95% confidence intervals; red dashed lines=reference AT (Outpatients: 7.0 °C for males, 5.0 °C for females, 8.5 °C for participants ≤5 , 5.0 °C for participants >5 to ≤14, 8.0 °C for participants >14 to ≤44, −2.5 °C for participants >44 to ≤65, −2.5 °C for participants >65; Inpatients: 7.3 °C for males, 9.0 °C for females, 7.5 °C for participants ≤5 , 9.5 °C for participants >5 to ≤14, 7.0 °C for participants >14 to ≤44, 7.0 °C for participants >44 to ≤65, 9.0 °C for participants >65); black dashed lines=moderate cold (2.5th, 2.4 °C) and moderate heat (75.0th, 32.1 °C), respectively.

**Fig. S8** **Lag-effects of moderate cold and moderate heat on daily outpatient visits for total respiratory diseases***

*This is a subgroup analysis which is stratified by gender, age. Moderate cold (2.4 °C) and moderate heat (32.1 °C) were the 2.5th and 75.0th percentiles of AT, respectively. The reference was the optimum AT of each group.
AT, apparent temperature; RR, relative risk.

**Fig. S9 Lag-effects of moderate cold and moderate heat on daily inpatient visits for total respiratory diseases***

*This is a subgroup analysis which is stratified by gender, age. Moderate cold (2.4 °C) and moderate heat (32.1 °C) were the 2.5th and 75.0th percentiles of AT, respectively. The reference was the optimum AT of each group.
AT, apparent temperature; RR, relative risk.

**Fig. S10 Exposure-response relationships between AT and risk of daily hospital visits for respiratory diseases: sensitivity analyses***
* Sensitivity analyses on the associations between AT and respiratory diseases with additional adjustment for sunshine time. We excluded data points where the ATs exceeded the 97.5th percentile while plotting the curve of LRTI in outpatients due to the limited amount of data available for those ATs.

AT, apparent temperature; RR, relative risk; COPD, chronic obstructive pulmonary disease; URTI, upper respiratory tract infection; LRTI, lower respiratory tract infection. Solid lines=mean RR (AT v the reference AT); shaded areas=95% confidence intervals; red dashed lines=reference AT (Outpatients: 5.4 °C for total respiratory diseases, 4.0 °C for influenza and pneumonia, 9.0 °C for URTI, 9.3 °C for LRTI, 4.0 °C for asthma, 4.0 °C for COPD; Inpatients: 7.8 °C for total respiratory diseases, 7.6 °C for influenza and pneumonia, 8.6 °C for URTI, 6.3 °C for LRTI, 10.0 °C for asthma, 9.0 °C for COPD); black dashed lines=moderate cold (2.5th, 2.4 °C) and moderate heat (75.0th, 32.1 °C), respectively.

**Fig. S11 Exposure-response relationships between AT and risk of daily hospital visits for respiratory diseases: sensitivity analyses***
* Sensitivity analyses on the associations between AT and respiratory diseases with additional adjustment for CO. We excluded data points where the ATs exceeded the 97.5th percentile while plotting the curve of LRTI in outpatients due to the limited amount of data available for those ATs.

AT, apparent temperature; RR, relative risk; COPD, chronic obstructive pulmonary disease; URTI, upper respiratory tract infection; LRTI, lower respiratory tract infection; CO, carbon monoxide. Solid lines=mean RR (AT v the reference AT); shaded areas=95% confidence intervals; red dashed lines=reference AT (Outpatients: 5.4 °C for total respiratory diseases, 4.0 °C for influenza and pneumonia, 9.0 °C for URTI, 10.3 °C for LRTI, 4.0 °C for asthma, 4.0 °C for COPD; Inpatients: 8.0 °C for total respiratory diseases, 7.5 °C for influenza and pneumonia, 9.2 °C for URTI, 7.7 °C for LRTI, 10.0 °C for asthma, 9.0 °C for COPD); black dashed lines=moderate cold (2.5th, 2.4 °C) and moderate heat (75.0th, 32.1 °C), respectively.

**Fig. S12 Exposure-response relationships between AT and risk of daily hospital visits for respiratory diseases: sensitivity analyses***
* Sensitivity analyses on the associations between AT and respiratory diseases with additional adjustment for O_3_. We excluded data points where the ATs exceeded the 97.5th percentile while plotting the curve of LRTI in outpatients due to the limited amount of data available for those ATs.

AT, apparent temperature; RR, relative risk; COPD, chronic obstructive pulmonary disease; URTI, upper respiratory tract infection; LRTI, lower respiratory tract infection; O_3_, ozone. Solid lines=mean RR (AT v the reference AT); shaded areas=95% confidence intervals; red dashed lines=reference AT (Outpatients: 5.0 °C for total respiratory diseases, 4.0 °C for influenza and pneumonia, 9.0 °C for URTI, 9.6 °C for LRTI, 4.0 °C for asthma, 4.0 °C for COPD; Inpatients: 7.7 °C for total respiratory diseases, 7.4 °C for influenza and pneumonia, 8.7 °C for URTI, 6.9 °C for LRTI, 10.0 °C for asthma, 9.0 °C for COPD); black dashed lines=moderate cold (2.5th, 2.4 °C) and moderate heat (75.0th, 32.1 °C), respectively.

**Fig. S13 Exposure-response relationships between AT and risk of daily hospital visits for respiratory diseases: sensitivity analyses***
* Sensitivity analyses on the associations between AT and respiratory diseases with additional adjustment for CO and O_3_. We excluded data points where the ATs exceeded the 97.5th percentile while plotting the curve of LRTI in outpatients due to the limited amount of data available for those ATs.

AT, apparent temperature; RR, relative risk; COPD, chronic obstructive pulmonary disease; URTI, upper respiratory tract infection; LRTI, lower respiratory tract infection; O_3_, ozone; CO, carbon monoxide. Solid lines=mean RR (AT v the reference AT); shaded areas=95% confidence intervals; red dashed lines=reference AT (Outpatients: 5.5 °C for total respiratory diseases, 4.0 °C for influenza and pneumonia, 9.0 °C for URTI, 10.3 °C for LRTI, 4.0 °C for asthma, 4.0 °C for COPD; Inpatients: 8.0 °C for total respiratory diseases, 7.5 °C for influenza and pneumonia, 9.1 °C for URTI, 7.8 °C for LRTI, 10.0 °C for asthma, 9.0 °C for COPD); black dashed lines=moderate cold (2.5th, 2.4 °C) and moderate heat (75.0th, 32.1 °C), respectively.

**Fig. S14 Exposure-response relationships between AT and risk of daily hospital visits for respiratory diseases: sensitivity analyses***
* Sensitivity analyses on the associations between AT and respiratory diseases using ambient temperature rather than AT as exposure. We excluded data points where the ATs exceeded the 97.5th percentile while plotting the curve of LRTI in outpatients due to the limited amount of data available for those ATs.

AT, apparent temperature; RR, relative risk; COPD, chronic obstructive pulmonary disease; URTI, upper respiratory tract infection; LRTI, lower respiratory tract infection. Solid lines=mean RR (ambient temperatures v the reference ambient temperature); shaded areas=95% confidence intervals; red dashed lines=reference ambient temperature (Outpatients: 9.0 °C for total respiratory diseases, 4.0 °C for influenza and pneumonia, 10.0 °C for URTI, 10.6 °C for LRTI, 4.0 °C for asthma, 4.0 °C for COPD; Inpatients: 10.2 °C for total respiratory diseases, 10.2 °C for influenza and pneumonia, 10.9 °C for URTI, 9.5 °C for LRTI, 12.0 °C for asthma, 9.3 °C for COPD); black dashed lines=moderate cold (2.5th, 5.4 °C) and moderate heat (75.0th, 27.8 °C), respectively.

**Table S1** **Summary statistics on meteorological variables and air pollutants in Ganzhou, 2016–2020**

| **Variables** | **Mean±SD** | **Minimum  value** | **P25** | **P50** | **P75** | **Maximum  value** |
| --- | --- | --- | --- | --- | --- | --- |
| Meteorological variables |  |  |  |  |  |  |
| T_max_ (°C) | 25.2±8.9 | 1.7 | 18.2 | 26.7 | 33.0 | 39.0 |
| T_min_ (°C) | 17.3±7.8 | −3.0 | 10.7 | 18.4 | 24.5 | 30.0 |
| Wind Speed (m/s) | 1.5±0.6 | 0.4 | 1.1 | 1.4 | 1.8 | 4.3 |
| Air pollutants |  |  |  |  |  |  |
| PM_2.5_ (μg/m^3^) | 37.4±20.9 | 6.0 | 23.0 | 33.0 | 47.0 | 184.0 |
| PM_10_ (μg/m^3^) | 60.1±33.6 | 11.0 | 36.0 | 52.0 | 75.0 | 246.0 |
| O_3_ (μg/m^3^) | 90.5±39.1 | 7.0 | 63.0 | 88.0 | 116.0 | 224.0 |
| CO (mg/m^3^) | 1.2±0.3 | 0.6 | 1.0 | 1.2 | 1.4 | 2.9 |
| NO_2_ (μg/m^3^) | 22.8±12.7 | 4.0 | 14.0 | 19.0 | 28.0 | 84.0 |
| SO_2_ (μg/m^3^) | 18.7±11.2 | 2.0 | 11.0 | 16.0 | 23.0 | 73.0 |

**Abbreviations:** SD, standard deviation; T_max_, daily maximum temperatures; T_min_, daily minimum temperatures. PM_2.5_, fine particulate matter; PM_10_, inhalable particles; O_3_, ozone; NO_2_, nitrogen dioxide; SO_2_, sulfur dioxide; CO, carbon monoxide.

**Table S2** **Relative risks of daily hospital visits for total respiratory diseases associated with non-optimum ATs***

| **Variables** | **Reference AT** | **Relative risk (95% CI)** | |
| --- | --- | --- | --- |
|  |  | **Moderate cold (2.5th, 2.4** °C**)** | **Moderate heat (75.0th, 32.1** °C**)** |
| Outpatients |  |  |  |
| Male | 7.0 | 1.017 (0.860, 1.203) | **1.428 (1.030, 1.980)** |
| Female | 5.0 | 1.016 (0.916, 1.127) | **1.734 (1.244, 2.417)** |
| Age, ≤5 years | 8.5 | 1.302 (0.935, 1.812) | **2.521 (1.598, 3.975) *** |
| Age, >5 to ≤14 years | 5.0 | 1.084 (0.857, 1.371) | **1.990 (1.017, 3.890)** |
| Age, >14 to ≤44 years | 8.0 | 1.097 (0.866, 1.391) | 1.359 (0.901, 2.049) |
| Age, >44 to ≤65 years | −2.5 | 1.156 (0.859, 1.557) | 1.394 (0.765, 2.541) |
| Age, >65 years | −2.5 | 1.163 (0.847, 1.597) | 1.264 (0.664, 2.406) |
| Inpatients |  |  |  |
| Male | 7.3 | 1.080 (0.954, 1.223) | **1.291 (1.008, 1.653)** |
| Female | 9.0 | 1.163 (0.952, 1.420) | 1.249 (0.897, 1.739) |
| Age, ≤5 years | 7.5 | 1.183 (0.996, 1.405) | **2.140 (1.448, 3.162) *** |
| Age, >5 to ≤14 years | 9.5 | 1.226 (0.796, 1.889) | 1.380 (0.652, 2.923) |
| Age, >14 to ≤44 years | 7.0 | 0.926 (0.643, 1.334) | 1.015 (0.519, 1.982) |
| Age, >44 to ≤65 years | 7.0 | 0.977 (0.802, 1.190) | 0.972 (0.672, 1.405) |
| Age, >65 years | 9.0 | 1.156 (0.941, 1.421) | 1.277 (0.922, 1.770) |

***** This is a subgroup analysis which is stratified by gender, age.

AT, apparent temperature; CI, confidence interval.

The statistically significant relative risks are highlighted in bold. ^*^ means *P* value < 0.05 obtained from Z-test for the difference among the relative risks derived from subgroup analyses.

**Table S3** **Relative risks of daily hospital visits for respiratory diseases associated with non-optimum ATs:** **sensitivity analyses***

| **Variables** | **Reference AT** | **Relative risk (95% CI)** | |
| --- | --- | --- | --- |
|  |  | **Moderate cold (2.5th, 2.4** °C**)** | **Moderate heat (75.0th, 32.1** °C**)** |
| Daily outpatient visits for respiratory diseases |  |  |  |
| Total | 5.4 | 1.019 (0.916, 1.132) | **1.595 (1.185, 2.148)** |
| Influenza and pneumonia | 4.0 | 0.967 (0.877, 1.065) | 1.645 (0.901, 3.004) |
| URTI | 9.0 | 1.269 (0.963, 1.672) | **1.711 (1.192, 2.455)** |
| LRTI | 9.3 | 1.471 (0.812, 2.663) | 2.157 (0.830, 5.607) |
| Asthma | 4.0 | 0.980 (0.910, 1.055) | 1.091 (0.702, 1.697) |
| COPD | 4.0 | 0.907 (0.800, 1.028) | 1.648 (0.878, 3.091) |
| Daily inpatient visits for respiratory diseases |  |  |  |
| Total | 7.8 | 1.104 (0.982, 1.240) | **1.280 (1.029, 1.592)** |
| Influenza and pneumonia | 7.6 | 1.093 (0.921, 1.298) | 1.185 (0.870, 1.615) |
| URTI | 8.6 | 1.186 (0.957, 1.469) | **2.043 (1.375, 3.035)** |
| LRTI | 6.3 | 1.058 (0.795, 1.408) | 1.841 (0.911, 3.719) |
| Asthma | 10.0 | 1.138 (0.789, 1.642) | 0.959 (0.533, 1.728) |
| COPD | 9.0 | 1.071 (0.879, 1.304) | 1.079 (0.781, 1.491) |

*These were the sensitivity analyses on the associations between AT and respiratory diseases with additionally adjustment for sunshine time.

**Abbreviations:** AT, apparent temperature; CI, confidence interval; COPD, chronic obstructive pulmonary disease; URTI, upper respiratory tract infection; LRTI, lower respiratory tract infection.

The statistically significant relative risks are highlighted in bold.

**Table S4 Relative risks of daily hospital visits for respiratory diseases associated with non-optimum ATs:** **sensitivity analyses***

| **Variables** | **Reference AT** | **Relative risk (95% CI)** | |
| --- | --- | --- | --- |
|  |  | **Moderate cold (2.5th, 2.4** °C**)** | **Moderate heat (75.0th, 32.1** °C**)** |
| Daily outpatient visits for respiratory diseases |  |  |  |
| Total | 5.4 | 1.023 (0.910, 1.150) | **1.527 (1.132, 2.059)** |
| Influenza and pneumonia | 4.0 | 0.962 (0.872, 1.061) | 1.618 (0.880, 2.975) |
| URTI | 9.0 | 1.260 (0.948, 1.674) | **1.586 (1.104, 2.279)** |
| LRTI | 10.3 | 1.742 (0.921, 3.293) | 1.969 (0.759, 5.109) |
| Asthma | 4.0 | 0.983 (0.912, 1.060) | 1.001 (0.638, 1.570) |
| COPD | 4.0 | 0.897 (0.789, 1.019) | 1.650 (0.874, 3.115) |
| Daily inpatient visits for respiratory diseases |  |  |  |
| Total | 8.0 | 1.112 (0.985, 1.255) | **1.253 (1.007, 1.559)** |
| Influenza and pneumonia | 7.5 | 1.094 (0.923, 1.297) | 1.175 (0.863, 1.601) |
| URTI | 9.2 | 1.237 (0.984, 1.555) | **1.940 (1.307, 2.879)** |
| LRTI | 7.7 | 1.132 (0.792, 1.617) | 1.640 (0.811, 3.316) |
| Asthma | 10.0 | 1.120 (0.771, 1.625) | 0.962 (0.534, 1.733) |
| COPD | 9.0 | 1.054 (0.863, 1.288) | 1.073 (0.776, 1.484) |

*These were the sensitivity analyses on the associations between AT and respiratory diseases with additionally adjustment for CO.

**Abbreviations:** AT, apparent temperature; CI, confidence interval; COPD, chronic obstructive pulmonary disease; URTI, upper respiratory tract infection; LRTI, lower respiratory tract infection; CO, carbon monoxide.

The statistically significant relative risks are highlighted in bold.

**Table S5 Relative risks of daily hospital visits for respiratory diseases associated with non-optimum ATs:** **sensitivity analyses***

| **Variables** | **Reference AT** | **Relative risk (95% CI)** | |
| --- | --- | --- | --- |
|  |  | **Moderate cold (2.5th, 2.4** °C**)** | **Moderate heat (75.0th, 32.1** °C**)** |
| Daily outpatient visits for respiratory diseases |  |  |  |
| Total | 5.0 | 1.012 (0.921, 1.112) | **1.55 (1.149, 2.092)** |
| Influenza and pneumonia | 4.0 | 0.963 (0.874, 1.061) | 1.654 (0.907, 3.016) |
| URTI | 9.0 | 1.261 (0.954, 1.665) | **1.693 (1.175, 2.44)** |
| LRTI | 9.6 | 1.575 (0.860, 2.883) | 1.929 (0.740, 5.031) |
| Asthma | 4.0 | 0.976 (0.906, 1.051) | 1.069 (0.688, 1.661) |
| COPD | 4.0 | 0.899 (0.793, 1.020) | 1.641 (0.875, 3.079) |
| Daily inpatient visits for respiratory diseases |  |  |  |
| Total | 7.7 | 1.097 (0.977, 1.231) | **1.254 (1.007, 1.562)** |
| Influenza and pneumonia | 7.4 | 1.085 (0.918, 1.281) | 1.176 (0.861, 1.607) |
| URTI | 8.7 | 1.189 (0.958, 1.476) | **1.964 (1.320, 2.924)** |
| LRTI | 6.9 | 1.087 (0.791, 1.494) | 1.841 (0.909, 3.730) |
| Asthma | 10.0 | 1.137 (0.789, 1.640) | 1.001 (0.554, 1.808) |
| COPD | 9.0 | 1.054 (0.865, 1.283) | 1.051 (0.759, 1.456) |

*These were the sensitivity analyses on the associations between AT and respiratory diseases with additionally adjustment for O_3_.

**Abbreviations:** AT, apparent temperature; CI, confidence interval; COPD, chronic obstructive pulmonary disease; URTI, upper respiratory tract infection; LRTI, lower respiratory tract infection; O_3_, ozone.

The statistically significant relative risks are highlighted in bold.

**Table S6 Relative risks of daily hospital visits for respiratory diseases associated with non-optimum ATs:** **sensitivity analyses***

| **Variables** | **Reference AT** | **Relative risk (95% CI)** | |
| --- | --- | --- | --- |
|  |  | **Moderate cold (2.5th, 2.4** °C**)** | **Moderate heat (75.0th, 32.1** °C**)** |
| Daily outpatient visits for respiratory diseases |  |  |  |
| Total | 5.5 | 1.019 (0.911, 1.139) | **1.503 (1.110, 2.034)** |
| Influenza and pneumonia | 4.0 | 0.962 (0.872, 1.061) | 1.581 (0.858, 2.912) |
| URTI | 9.0 | 1.266 (0.952, 1.683) | **1.656 (1.143, 2.400)** |
| LRTI | 10.3 | 1.743 (0.921, 3.299) | 1.857 (0.704, 4.896) |
| asthma | 4.0 | 0.978 (0.907, 1.055) | 0.988 (0.629, 1.550) |
| COPD | 4.0 | 0.895 (0.787, 1.017) | 1.657 (0.875, 3.137) |
| Daily inpatient visits for respiratory diseases |  |  |  |
| Total | 8.0 | 1.107 (0.982, 1.248) | 1.226 (0.983, 1.530) |
| Influenza and pneumonia | 7.5 | 1.090 (0.919, 1.293) | 1.139 (0.831, 1.561) |
| URTI | 9.1 | 1.227 (0.977, 1.539) | **1.894 (1.270, 2.824)** |
| LRTI | 7.8 | 1.139 (0.793, 1.636) | 1.674 (0.822, 3.410) |
| Asthma | 10.0 | 1.130 (0.778, 1.641) | 0.992 (0.548, 1.798) |
| COPD | 9.0 | 1.048 (0.858, 1.280) | 1.047 (0.754, 1.452) |

*These were the sensitivity analyses on the associations between AT and respiratory diseases with additionally adjustment for CO and O_3_.

**Abbreviations:** AT, apparent temperature; CI, confidence interval; COPD, chronic obstructive pulmonary disease; URTI, upper respiratory tract infection; LRTI, lower respiratory tract infection; O_3_, ozone; CO, carbon monoxide.

The statistically significant relative risks are highlighted in bold.

**Table S7 Relative risks of daily hospital visits for respiratory diseases associated with non-optimum ambient temperatures:** **sensitivity analyses***

| **Variables** | **Reference temperature** | **Relative risk (95% CI)** | |
| --- | --- | --- | --- |
|  |  | **Moderate cold (2.5th, 5.4** °C**)** | **Moderate heat (75.0th, 27.8** °C**)** |
| Daily outpatient visits for respiratory diseases |  |  |  |
| Total | 9.0 | 1.034 (0.897, 1.192) | **1.433 (1.081, 1.899)** |
| Influenza and pneumonia | 4.0 | 1.012 (0.899, 1.139) | 1.713 (0.926, 3.168) |
| URTI | 10.0 | **1.314 (1.029, 1.678)** | **1.632 (1.166, 2.283)** |
| LRTI | 10.6 | 1.400 (0.810, 2.419) | 1.683 (0.690, 4.105) |
| Asthma | 4.0 | 1.015 (0.927, 1.111) | 0.976 (0.614, 1.551) |
| COPD | 4.0 | 1.121 (0.963, 1.305) | 1.886 (0.950, 3.745) |
| Daily inpatient visits for respiratory diseases |  |  |  |
| Total | 10.2 | **1.148 (1.018, 1.296)** | **1.366 (1.108, 1.684)** |
| Influenza and pneumonia | 10.2 | 1.163 (0.972, 1.393) | 1.266 (0.944, 1.698) |
| URTI | 10.9 | **1.263 (1.013, 1.575)** | **1.880 (1.289, 2.741)** |
| LRTI | 9.5 | 1.161 (0.830, 1.624) | **2.132 (1.094, 4.153)** |
| Asthma | 12.0 | 1.186 (0.824, 1.708) | 1.078 (0.615, 1.888) |
| COPD | 9.3 | 1.068 (0.910, 1.253) | 1.234 (0.900, 1.691) |

*These were the sensitivity analyses on the associations between temperatures and respiratory diseases using ambient temperature rather than AT as exposure.

**Abbreviations:** AT, apparent temperature; CI, confidence interval; COPD, chronic obstructive pulmonary disease; URTI, upper respiratory tract infection; LRTI, lower respiratory tract infection.

The statistically significant relative risks are highlighted in bold.

**Fig. S1
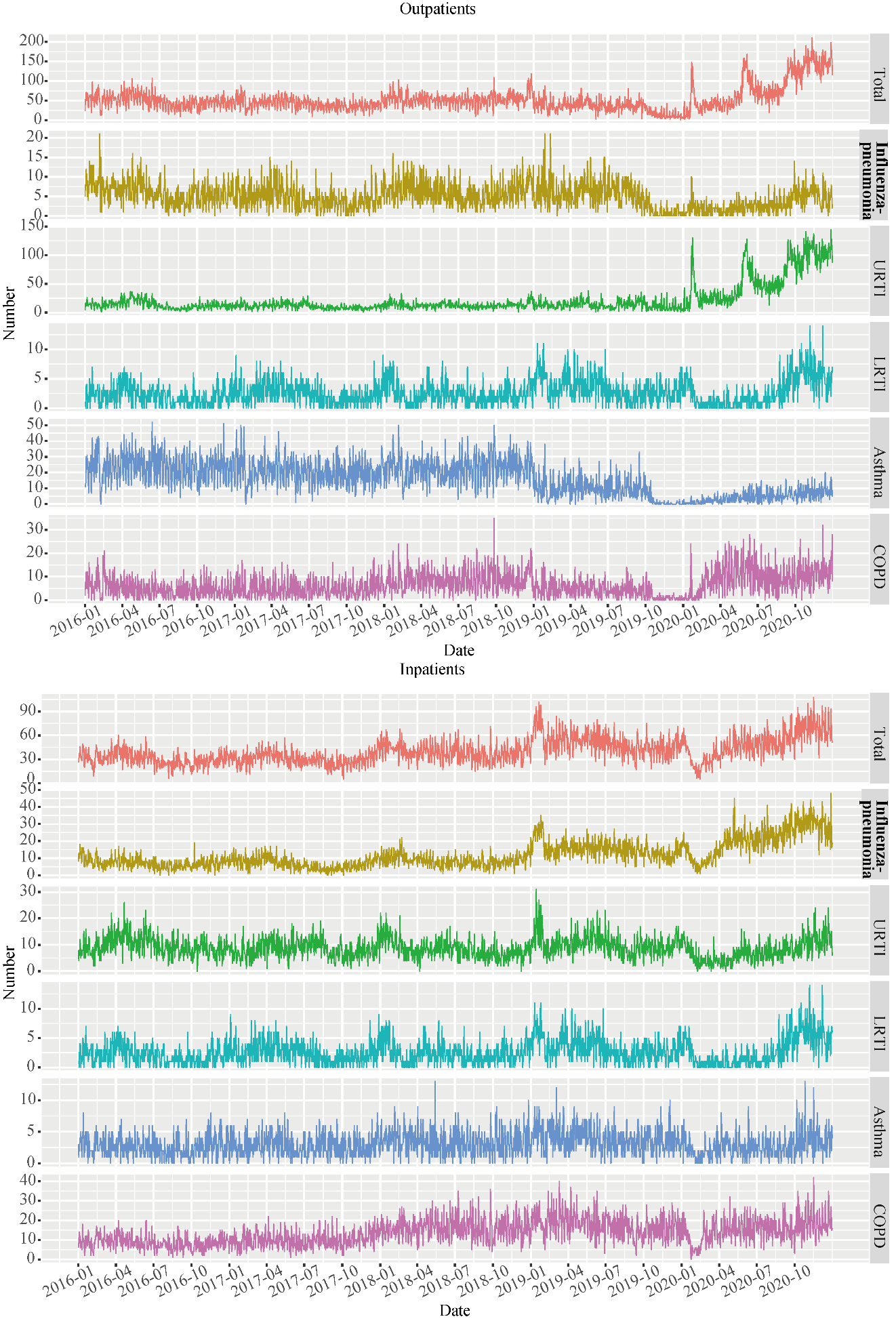
**

**Fig. S2**

**
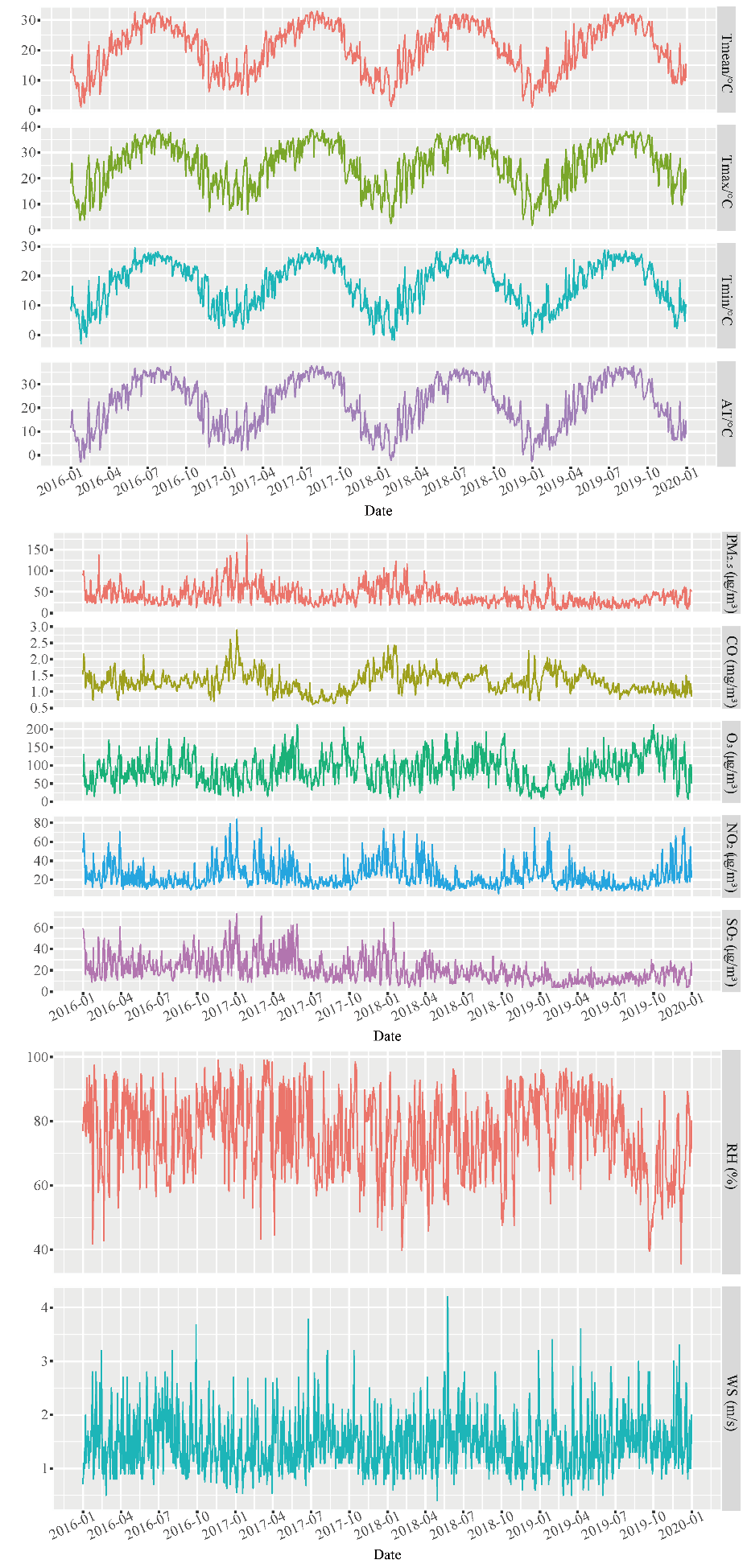
**

**Fig. S3**
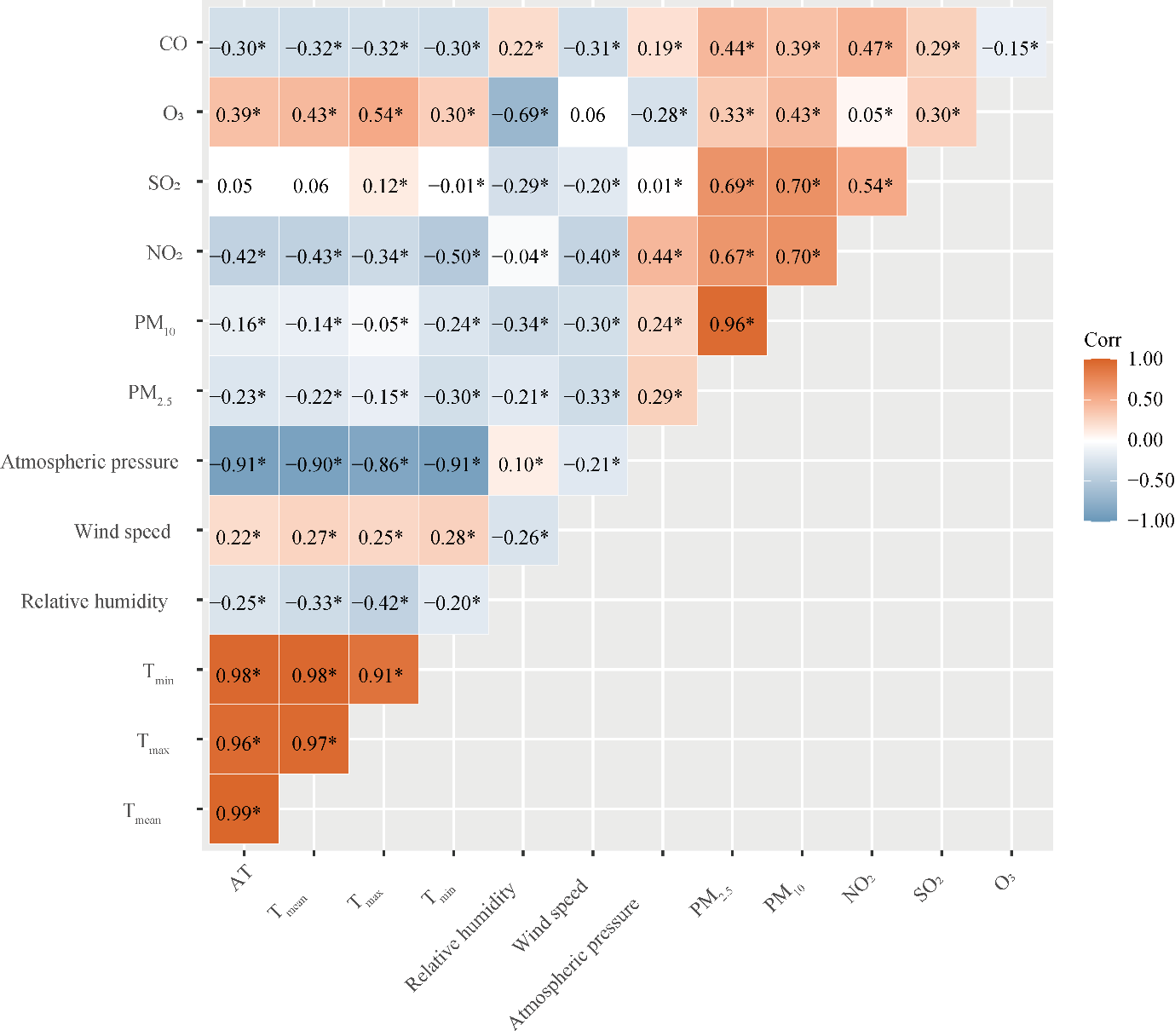


**Fig. S4**
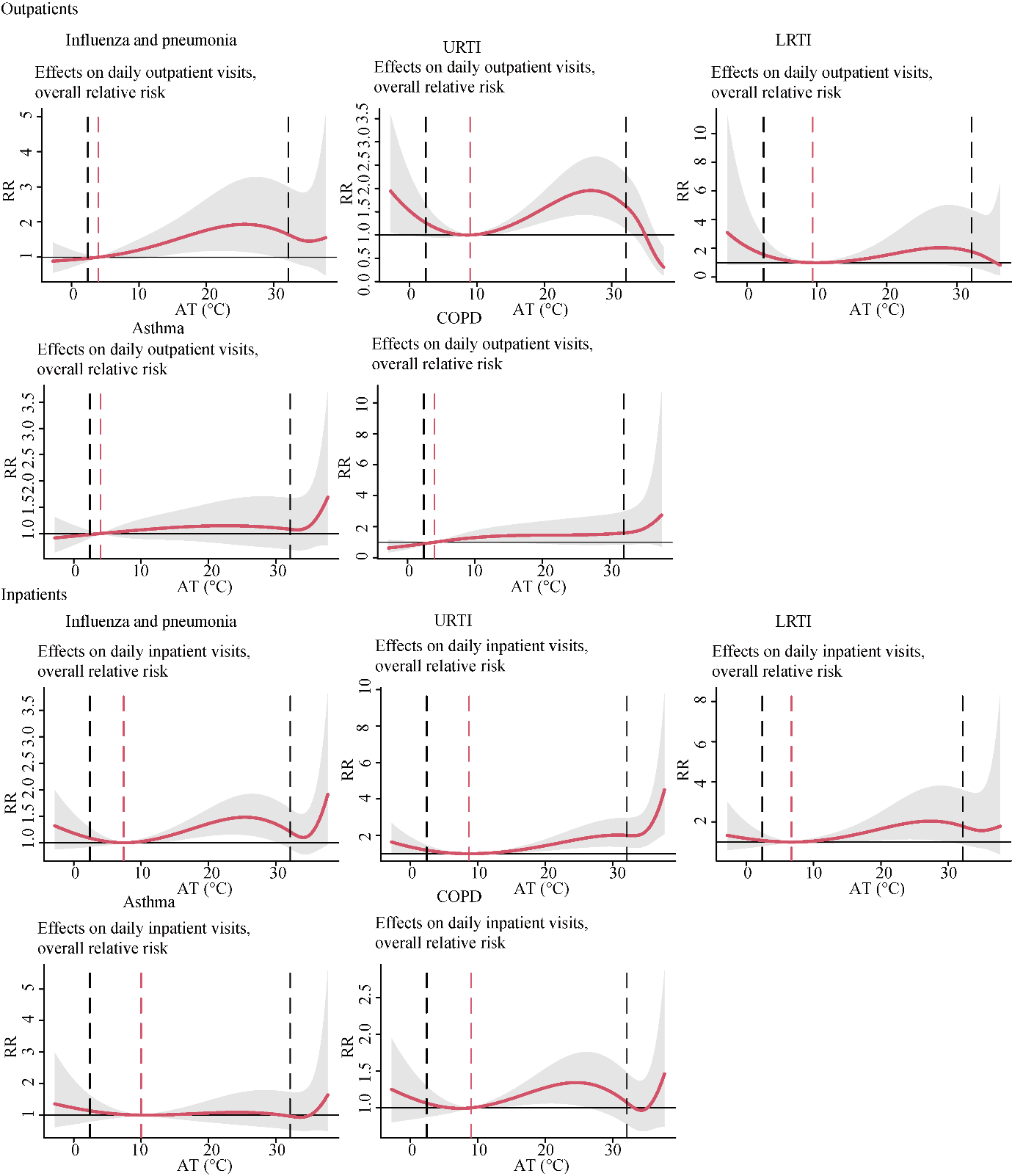


**Fig. S5**
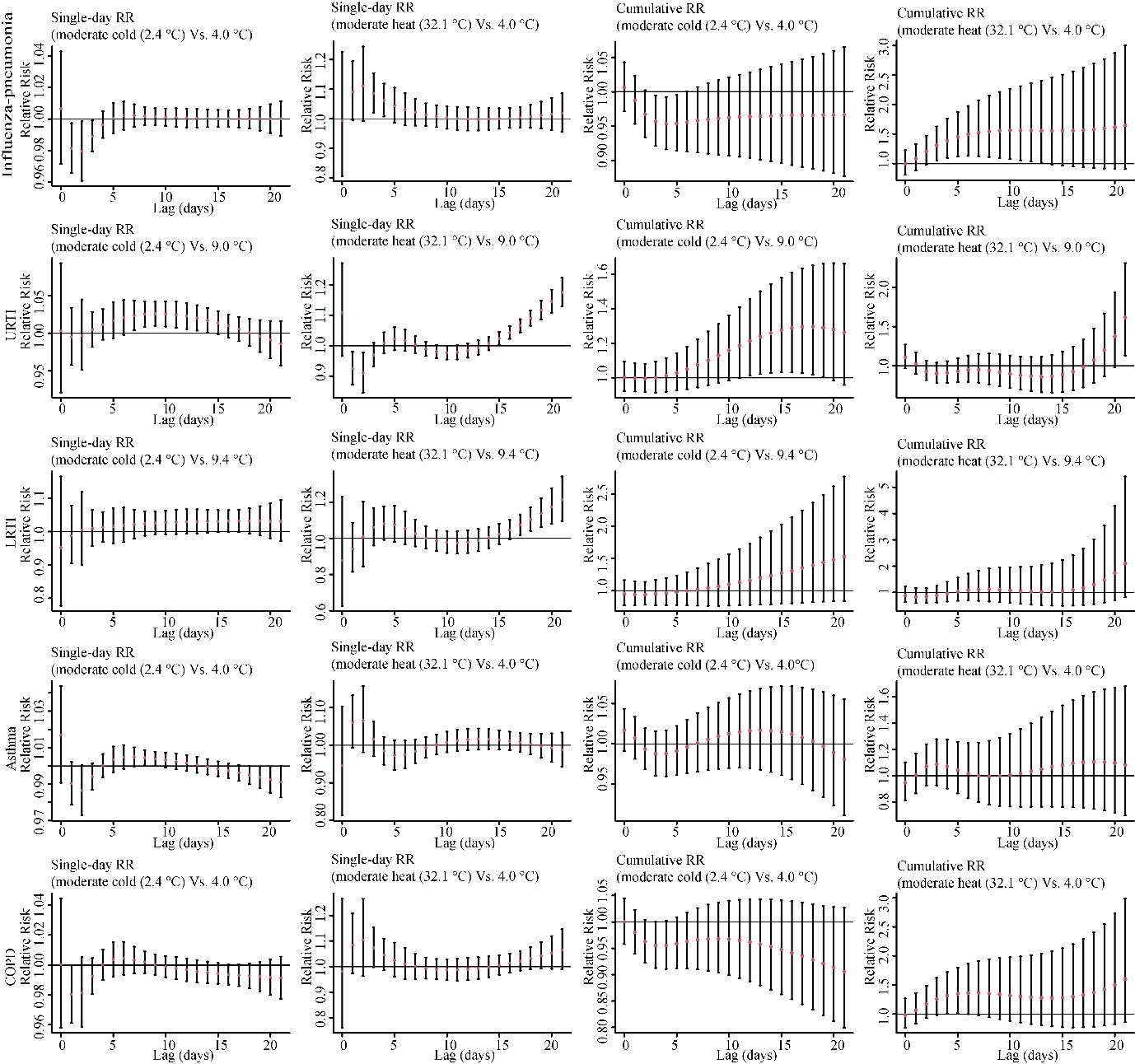


**Fig. S6**
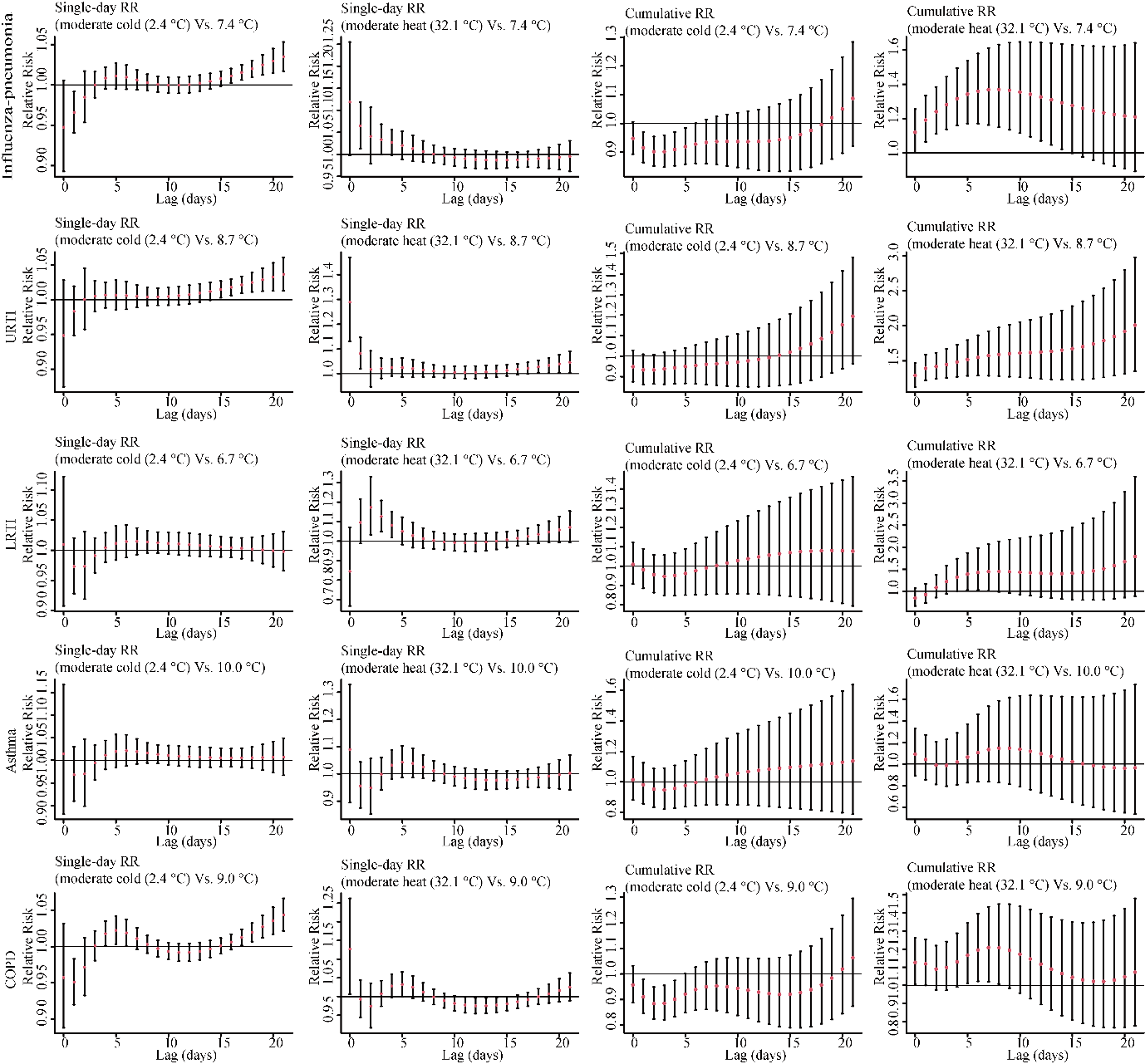


**Fig. S7**
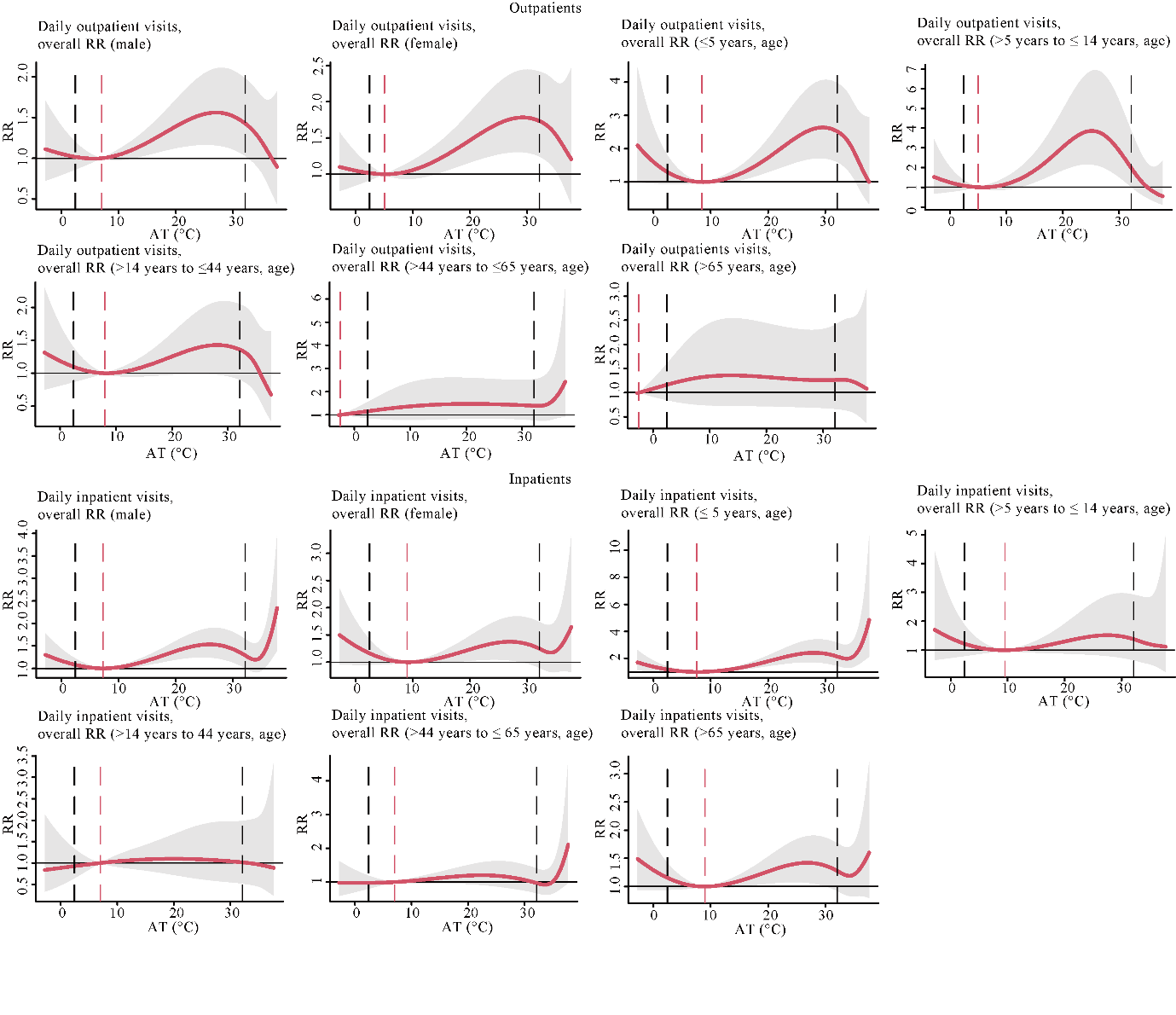


**Fig. S8**
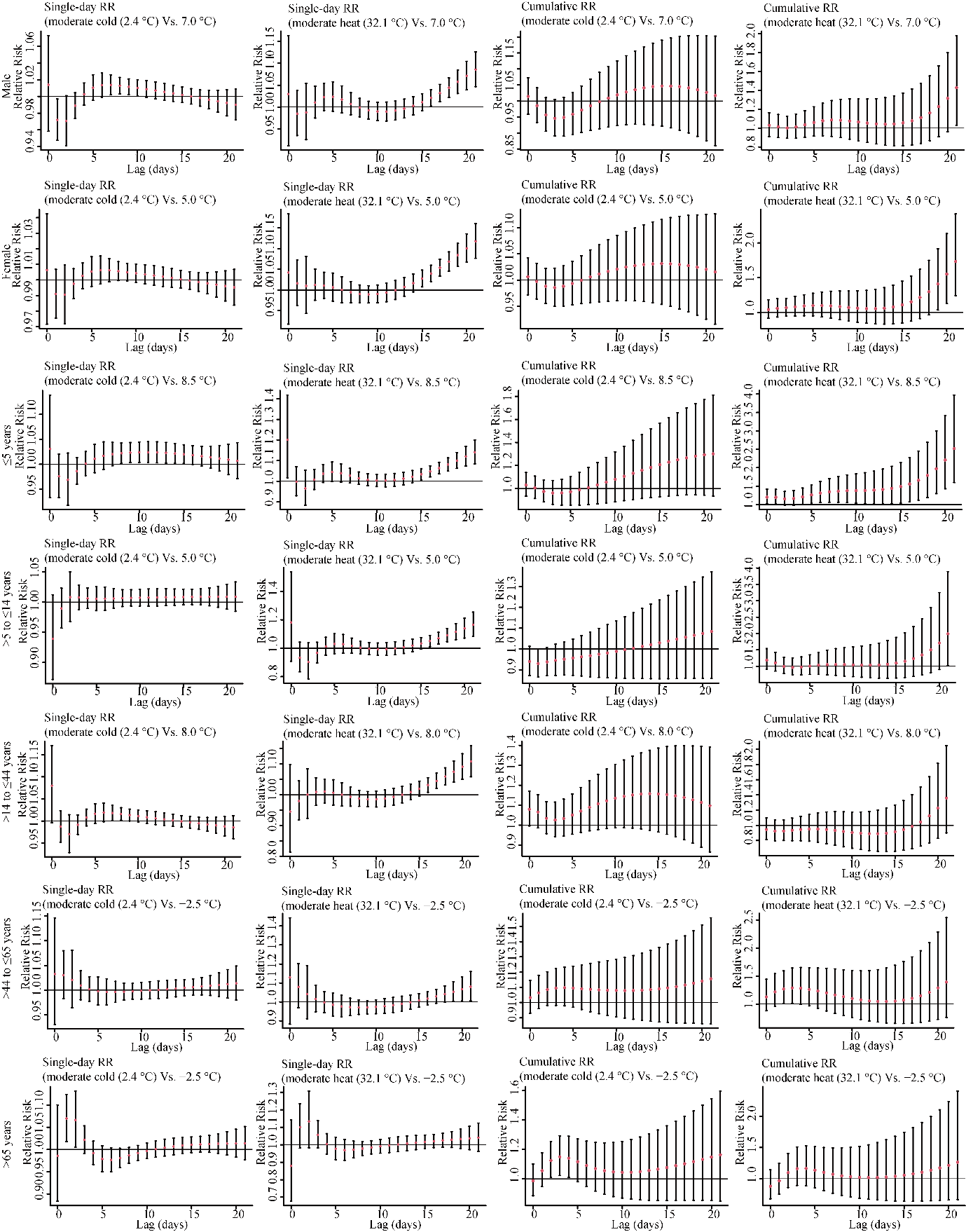


**Fig. S9**
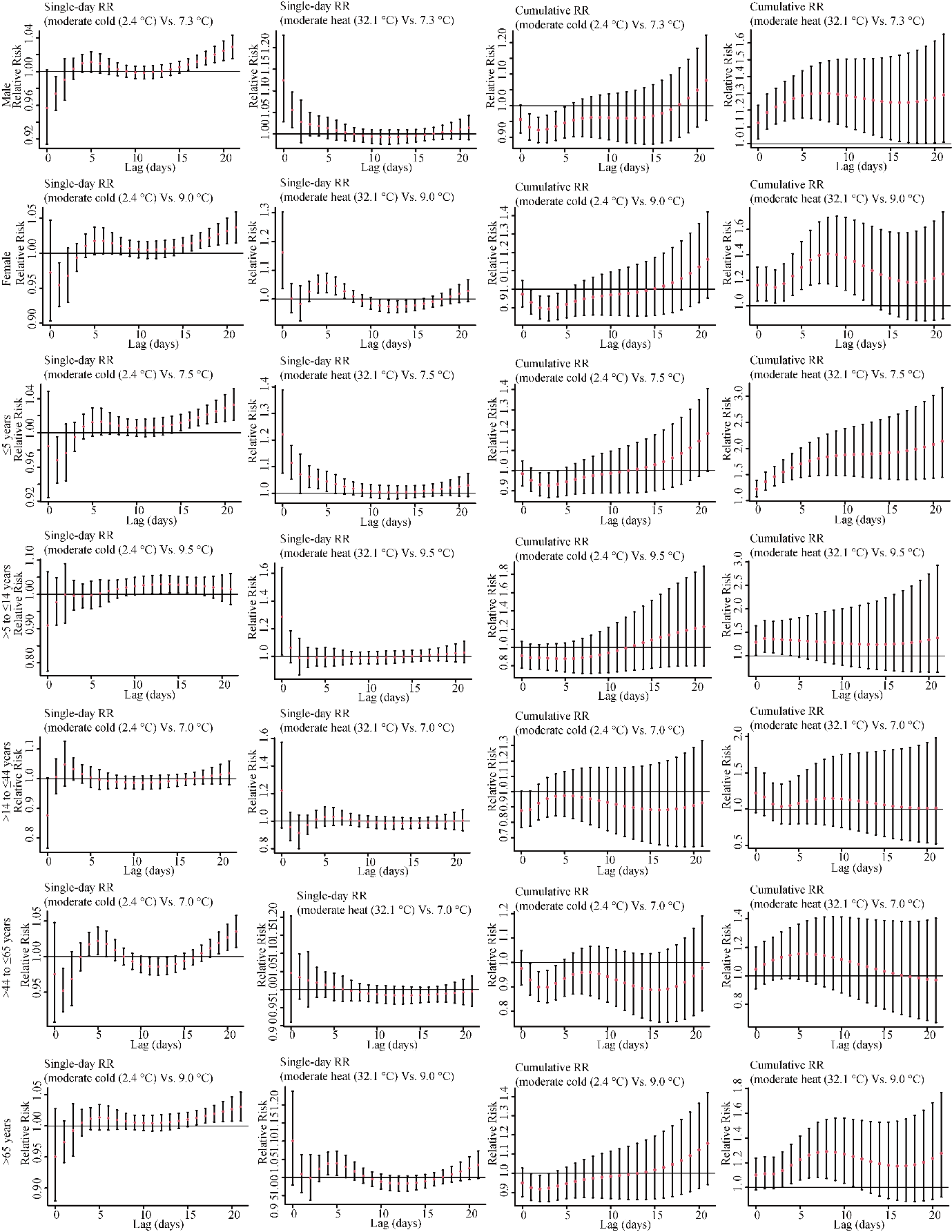


**Fig. S10**
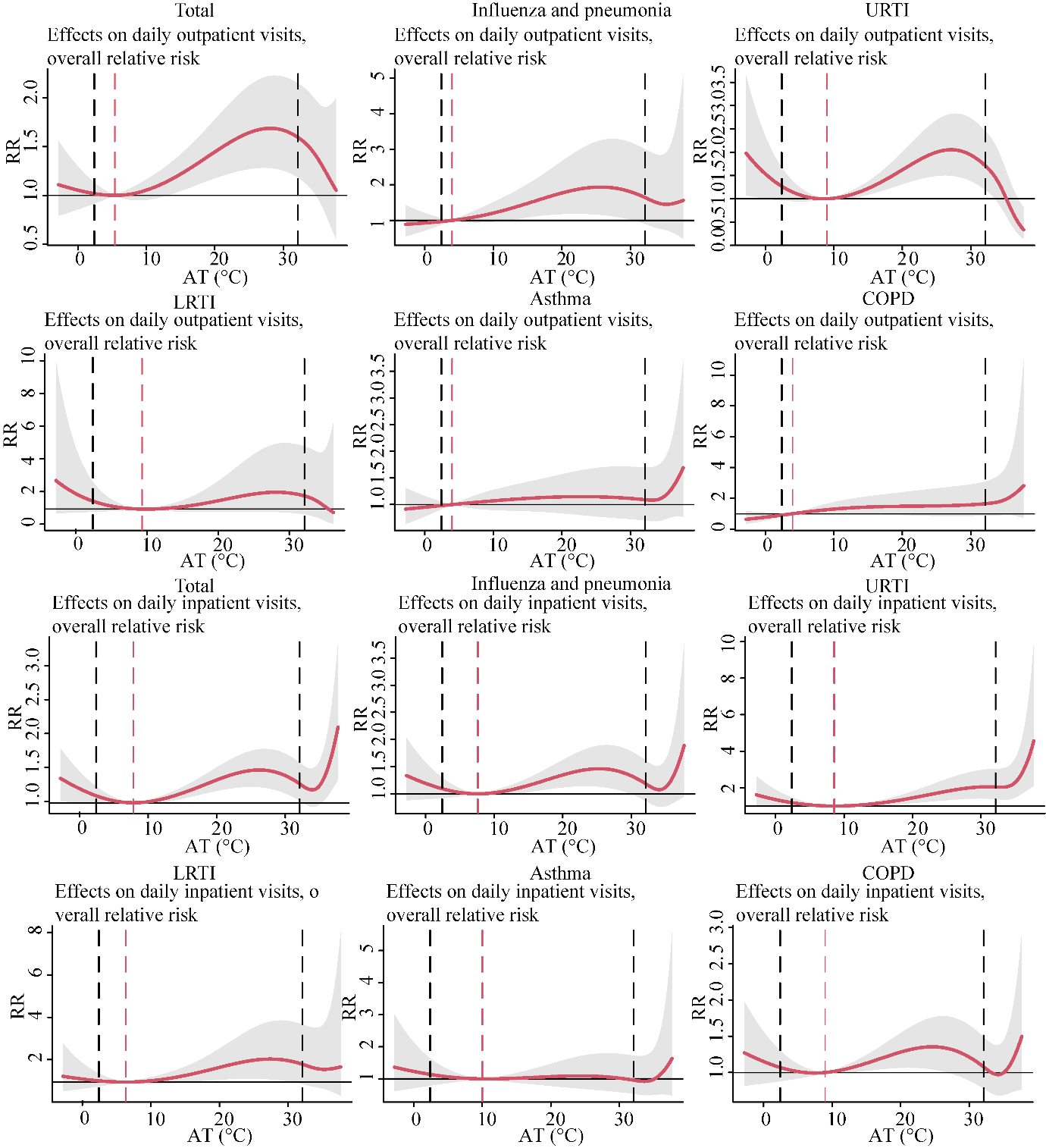


**Fig. S11**
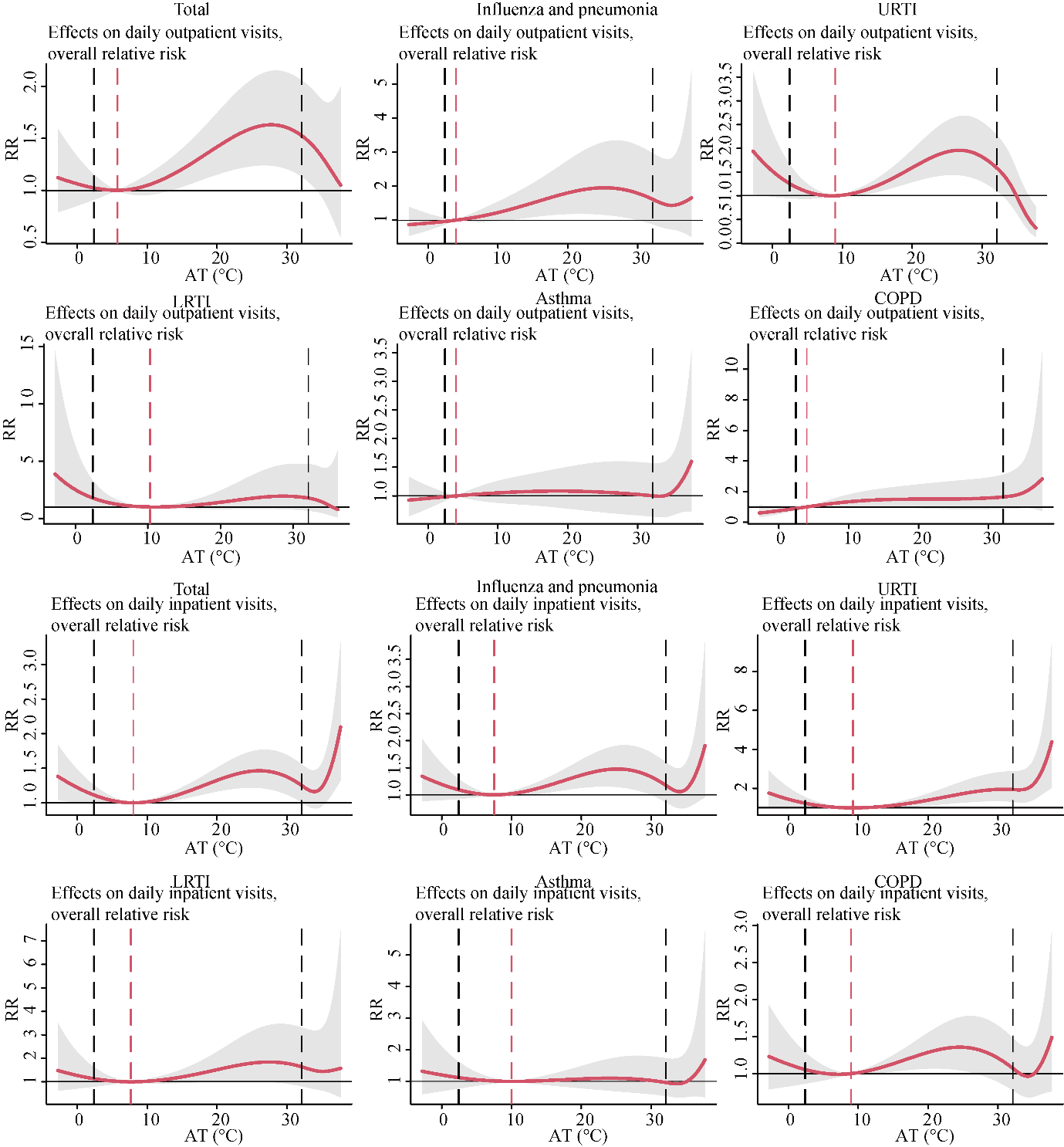


**Fig. S12**
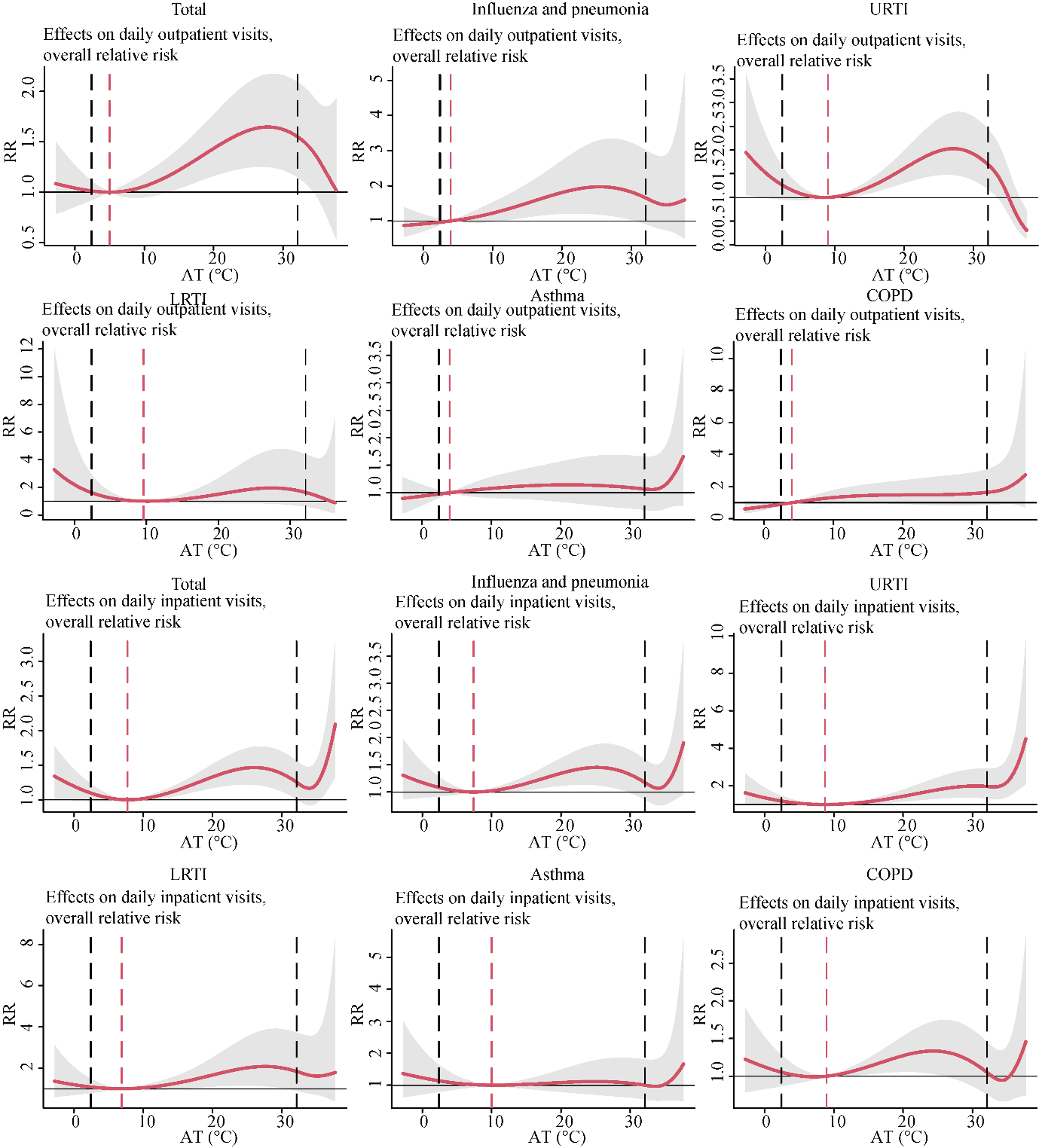


**Fig. S13**
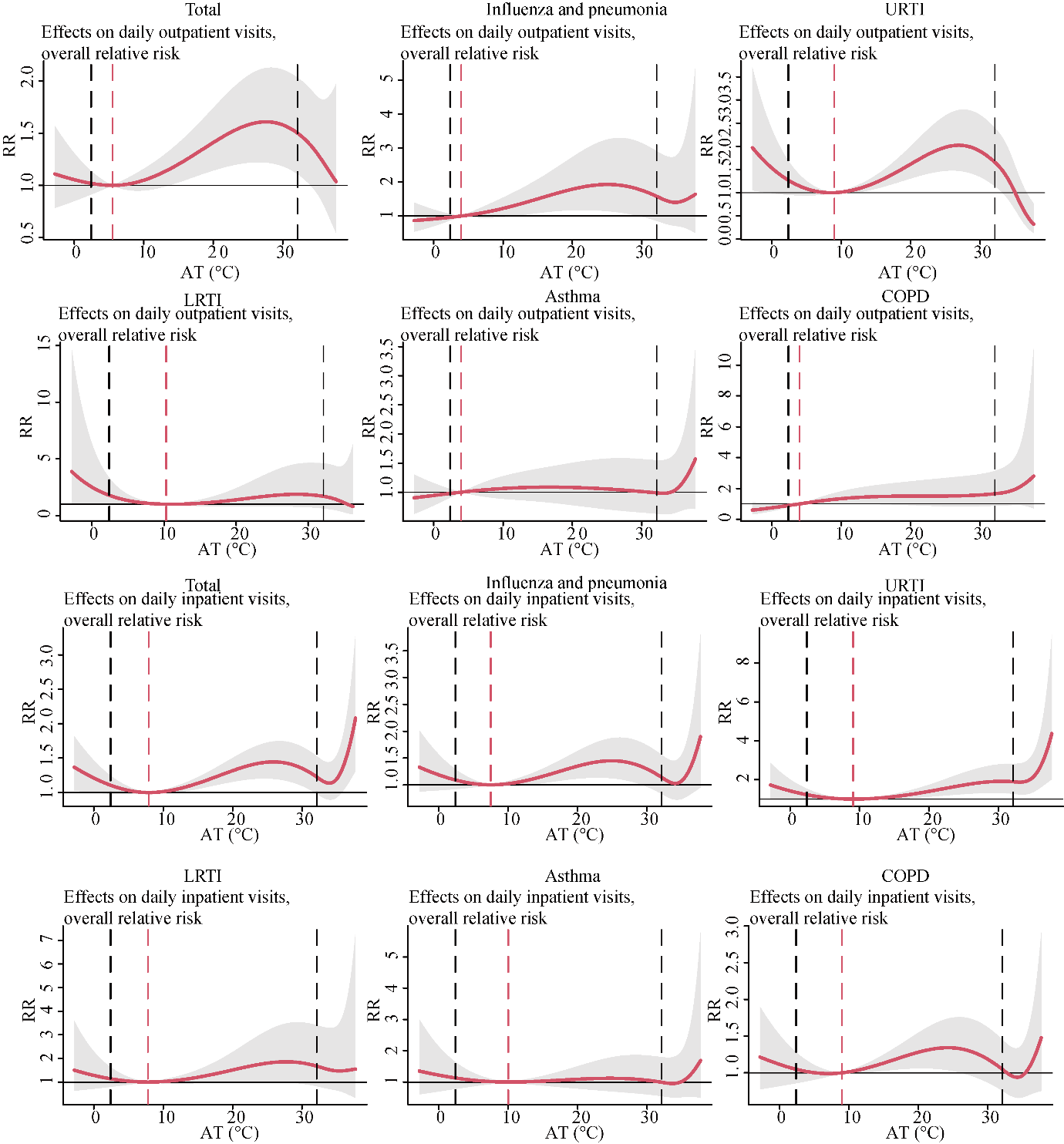


**Fig. S14**
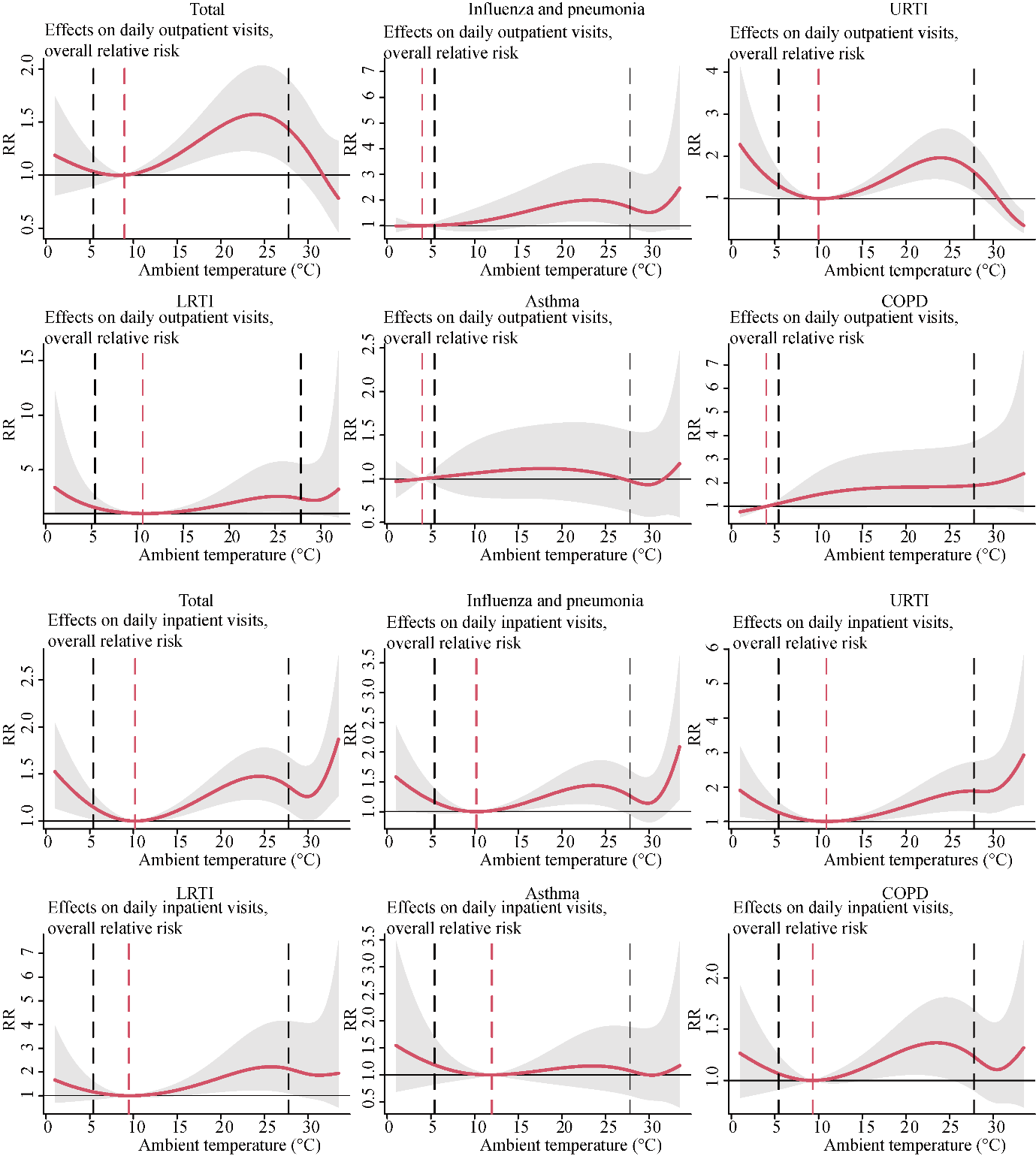

Supplement: Supplementary file 1 — Additional file 1: Fig. S1 Time-series distributions of daily hospital visits for cause-specific respiratory diseases in Ganzhou, 2016–2020. Fig. S2 Time-series distributions of meteorological variables and concentrations of air pollutants in Ganzhou, 2016–2020. Fig. S3 Spearman’s correlation of air pollutants and meteorological variables. Fig. S4 Exposure-response relationships between AT and risk of daily hospital visits for five cause-specific respiratory diseases*. Fig. S5 Lag-effects of moderate cold and moderate heat on daily outpatient visits for cause-specific respiratory diseases*. Fig. S6 Lag-effects of moderate cold and moderate heat on daily inpatient visits for cause-specific respiratory diseases*. Fig. S7 Exposure-response relationships between AT and risk of daily hospital visits for total respiratory diseases*. Fig. S8 Lag-effects of moderate cold and moderate heat on daily outpatient visits for total respiratory diseases*. Fig. S9 Lag-effects of moderate cold and moderate heat on daily inpatient visits for total respiratory diseases*. Fig. S10 Exposure-response relationships between AT and risk of daily hospital visits for respiratory diseases: sensitivity analyses*. Fig. S11 Exposure-response relationships between AT and risk of daily hospital visits for respiratory diseases: sensitivity analyses*. Fig. S12 Exposure-response relationships between AT and risk of daily hospital visits for respiratory diseases: sensitivity analyses*. Fig. S13 Exposure-response relationships between AT and risk of daily hospital visits for respiratory diseases: sensitivity analyses*. Fig. S14 Exposure-response relationships between AT and risk of daily hospital visits for respiratory diseases: sensitivity analyses*. Table S1 Summary statistics on meteorological variables and air pollutants in Ganzhou, 2016–2020. Table S2 Relative risks of daily hospital visits for total respiratory diseases associated with non-optimum ATs*. Table S3 Relative risks of daily hospital vis [file ehpm-29-020-s001.docx]
